# Artificial intelligence augmented tutoring vs expert instruction on learning simulated general surgical skills: a systematic review and meta-analysis

**DOI:** 10.1186/s12909-026-09606-9

**Published:** 2026-06-10

**Authors:** Fouad Hanna, Mahmoud Mohamed Gad, Mohamed Bassiouny Fouad Helmy, Mohamed Mostafa Eisa, Mohamed Wael Z. Omran, Muhammad Youssef, Ahmed Youssef Hassan, Abdelrhman Waleed Kotb, Mariam A. Abusalah, Abdelrahman El-Helbawy, Mina Gamil Zekri Basta, Abd-Elfattah Kalmoush, Mahmoud Diaa Hindawi

**Affiliations:** 1 Faculty of Medicine, Cairo University, Khalfawy Street, Cairo, Giza, 11511 Egypt; 2 Faculty of Medicine, Mansoura University - Mansoura University, Mansoura, Egypt; 3 Faculty of Medicine, Al-Azhar University, Cairo, Egypt; 4 Faculty of Medicine, Tanta University, Tanta, Egypt; 5 Kafr El-Sheikh University, Kafrelsheikh, Egypt; 6 Faculty of Medicine, Al Quds University, Abu Dis, Palestine; 7 Faculty of Medicine, Helwan University, Cairo, Egypt; 8 Department of Vascular surgery, Faculty of Medicine, Ain Shams University, Cairo, Egypt; 9 General Surgery Department, Al-Azhar University, Cairo, Egypt

**Keywords:** Artificial Intelligence, Surgical Education, Meta-Analysis, Intelligent Tutoring Systems, Skill Assessment, Medical Training

## Abstract

**Background:**

Surgical training suffers from a global deficit; 5 billion people lack access to safe surgery, with an estimated 143 million additional procedures needed annually. Traditional surgical education, constrained by the apprenticeship model, faces critical limitations in standardization and scalability, particularly in low- and middle-income countries where expert mentors are scarce. AI-augmented tutoring systems represent a potentially transformative solution. This systematic review and meta-analysis were conducted to address that evidence gap.

**Methods:**

Following PRISMA 2020 guidelines, we systematically searched major databases for trials comparing AI tutoring with expert instruction. Primary outcomes were performance (Intelligent Continuous Expertise Monitoring System [ICEMS] score) and skill acquisition (Objective Structured Assessment of Technical Skills [OSATS] score). Cognitive load was a secondary outcome, measured using the Mental Effort Scale (MES) and Cognitive Load Index (CLI).

**Results:**

Our search yielded 40 studies for narrative synthesis. Four studies (3 RCTs and 1 pilot prospective study), encompassing a total of 268 participants, were included in the meta-analysis. AI tutoring showed a small, statistically significant improvement in expert-rated OSATS scores (MD 0.20; 95% CI, 0.01 to 0.39) with no heterogeneity (I2 = 0%) but with low certainty. The AI group reported a significantly higher extraneous cognitive load (MD 0.23; *p* = 0.01). No significant difference was found in ICEMS scores.

**Conclusion:**

AI tutoring systems demonstrated comparable effectiveness to expert instructors in simulated surgical skill acquisition. The small OSATS advantage (0.20 points) is of uncertain clinical significance, falling below commonly published competency cut-points for meaningful change on global rating scales, and is based on low-certainty evidence driven by a single high-risk-of-bias study. AI tutoring imposed a higher extraneous cognitive load. These findings do not support replacing human instructors with AI. Instead, the evidence supports a hybrid model, though this itself requires rigorous empirical validation.

**Graphical Abstract:**

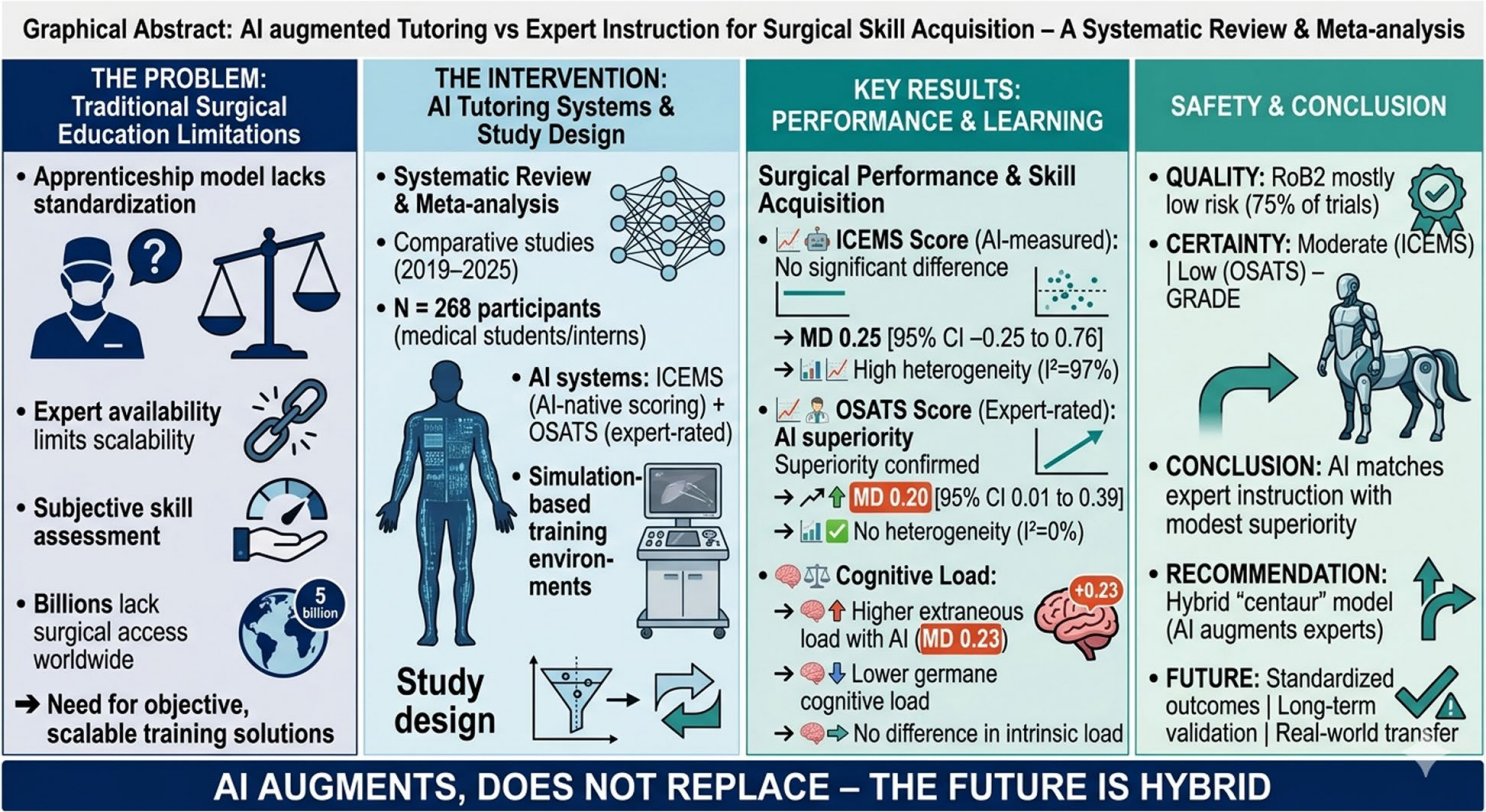

**Supplementary Information:**

The online version contains supplementary material available at 10.1186/s12909-026-09606-9.

## Introduction

Surgical education is essential for learning complex bimanual surgical skills [1, 2]. Wide differences in surgical skills among surgeons are associated with a higher risk of intraoperative and postoperative surgical complications [3]. Traditionally, surgical education has relied on the apprenticeship model, famously summarized as "see one, do one, teach one" [4]. While this method has produced generations of skilled surgeons, it is inherently limited by the availability of expert mentors and the high-pressure environment of the operating room, which restricts opportunities for hands-on practice, especially for novice trainees [5]. Conventional surgical training models are subjective and lack standardization, depending mainly on expert observation and judgment without quantitative, measurable data [6, 7]. Standardized surgical training programs are essential to enhance trainee skills and improve patient outcomes [8].

The integration of artificial intelligence (AI) into surgical education has accelerated in recent years, driven in part by reduced intraoperative teaching opportunities during the COVID-19 pandemic [9]. Within surgical training specifically, AI technologies can address limitations of traditional systems by providing objective, data-driven performance assessment and reducing evaluator variability [10–14].

In this review, an AI tutoring system (also termed an "intelligent tutoring system") is defined as an educational platform driven by computer algorithms that: (1) integrates performance assessment with personalized, goal-oriented feedback; and (2) is designed to guide surgical skill acquisition in a training context [15]. These systems use machine learning algorithms to classify learner performance on quantifiable metrics and deliver tailored audiovisual instruction, distinguishing them from broader AI applications such as computer vision for intraoperative anatomy recognition, natural language processing for operative note generation, or AI-assisted scheduling tools. By integrating virtual reality (VR) and augmented reality (AR) simulators, AI tutoring systems can create realistic environments in which trainees can repeatedly practice procedures without risk to patients [16, 17].

The ICEMS, an example of an AI tutoring system, can analyze and measure performance objectively. ICEMS can monitor the performance of the surgeons with millisecond accuracy and evaluate instrument handling, efficiency of motion, and tissue interaction [18]. In a randomized controlled trial assessing VR, medical students who trained with VR had better performance than peers with standard training [19]. However, a conceptual framework for hybrid human–AI collaboration has proposed that combining AI capabilities with human expertise may yield superior outcomes across complex professional tasks [20].

This systematic review and meta-analysis was designed to evaluate the role of AI tutoring systems as an augmentation of expert-led surgical instruction, rather than as a replacement for the human educator. We do not treat AI and the expert as competing entities; rather, we ask whether the integration of AI feedback into a training pathway — either as a parallel tutor or as a quantitative substrate that informs expert instruction — adds measurable value to skill acquisition over expert instruction alone. Accordingly, we provide a narrative synthesis of the broader landscape of AI applications in surgical education and a meta-analysis of trials that quantify the effect of AI tutoring on standardised performance outcomes (ICEMS, OSATS) and cognitive load.

## Methods and materials

### Guidelines and ethics

This systematic review and meta-analysis were conducted in accordance with a registered protocol on PROSPERO (CRD420251131271) [21] and followed the PRISMA (Preferred Reporting Items for Systematic Reviews and Meta-Analyses) guidelines throughout to ensure methodological rigor and transparency [22]. Since only previously published studies were used, Institutional Review Board (IRB) approval was not required. A completed PRISMA 2020 27-item checklist is provided as Electronic Supplementary Material 2.

### Literature search

A systematic literature search was conducted across multiple electronic databases, including PubMed, Scopus, Web of Science, Embase, Ovid, and Cochrane from their inception to August 2025. The search was supplemented by manual screening of reference lists from relevant articles (citation analysis) and other manual search methods to identify additional studies, and a librarian reviewed the search strategy. The search strategy was designed to be broad to capture all relevant literature and combined keywords related to artificial intelligence, surgical education, and skill assessment. The following search query was adapted for each database: ((Artificial Intelligence OR AI OR artificial intelligence OR ChatGPT OR intelligent tutoring system)) AND ((Education OR Teaching OR Tutoring OR Instruction OR Teaching OR Education OR Learning OR Training OR Computer-Assisted Instructions)) AND ((Surgical Procedures Operative OR surgical skill OR surgical train OR surgical educat OR surgical simulation OR procedural skill OR surgical performance OR operative skill)). (Electronic Supplementary Material 1).

We acknowledge that searching both MEDLINE (via Ovid) and PubMed resulted in overlapping records. This was a deliberate decision to maximize search sensitivity, as PubMed includes some records not yet indexed in MEDLINE (e.g., ahead-of-print articles). All duplicates were identified and removed during the deduplication phase using the Rayyan platform, and this did not affect the final set of included studies.

### Study selection

This review focused on general surgery and simulated surgical skill training. The definition of "AI-augmented tutoring," as operationalized in this review, refers to any computational system (including machine learning or deep learning platforms) that: (1) analyzes trainee performance data from a simulator; (2) generates automated, personalized feedback; and (3) is designed to guide skill acquisition in a training context. This definition encompasses real-time feedback systems, post-hoc AI coaching reports, and hybrid AI-plus-expert platforms. Studies implementing AI only for skill classification, phase recognition, or intraoperative guidance — without a tutoring/feedback component for learner development — were included in the narrative synthesis but excluded from the meta-analysis.

To guide our study selection, we applied the PICOS framework:

- Population (P): Medical trainees (e.g., students, residents) and surgeons engaged in simulation-based surgical skill training.
- Intervention (I): An AI tutoring system, defined as an educational platform driven by computer algorithms that (1) analyses trainee performance data, (2) generates automated, personalised feedback, and (3) is designed to guide surgical skill acquisition. The intervention encompasses two integration models: an AI-substitutive model, in which the AI delivers feedback directly to the trainee in place of an expert; and an AI-augmentative model, in which AI-derived performance data is used by, or supplements the work of, a human expert educator.
- Comparator (C): Expert-led instruction without AI support. In AI-substitutive trials, the comparator is expert instruction delivered as a parallel arm (AI vs Expert). In AI-augmentative trials, the comparator is expert instruction without AI (AI + Expert vs Expert).
- Outcomes (O): Surgical performance (ICEMS Expertise Score), skill acquisition and transfer (OSATS), and cognitive load.
- Study Design (S): Randomized controlled trials (RCTs), non-randomized controlled trials NCTs), and observational studies that included a comparator group.

Inclusion criteria: Studies were included if they (a) enrolled medical trainees or surgeons, (b) evaluated an AI tutoring system as defined above, (c) included a comparator group receiving expert instruction, and (d) reported at least one quantifiable performance outcome.

Exclusion criteria: Studies were excluded if they met any of the following criteria: (1) Animal or in vitro cell culture studies. (2) Abstracts, reviews, editorials, commentaries, case studies, case series, or literature with incomplete or inaccessible original data. (3) Studies focused on specialties other than general surgery. (4) Studies that did not include a direct comparison between an AI-based intervention and an expert instruction comparator.

### Screening and data extraction

Eight reviewers participated in the screening process. To ensure consistency across this team, the following procedures were implemented: (1) all reviewers completed a calibration exercise using a pilot set of 20 articles, with discrepancies discussed to establish shared decision rules prior to formal screening; (2) screening was conducted in pairs, with each pair independently reviewing assigned records using the Rayyan platform [23]; (3) disagreements within pairs were resolved by discussion, and unresolved conflicts were adjudicated by a senior reviewer (F.H.); (4) inter-rater agreement was assessed using percent agreement calculated across all paired screening decisions during title/abstract screening (n = 15,594 records), with average agreement exceeding 90% across all reviewer pairs. Formal Cohen's kappa was not calculated, which is acknowledged as a limitation. Data extraction was performed independently by two reviewers using a standardized data extraction form. The extracted information included: first author, year of publication, study design, sample size, participant characteristics (e.g., training level), details of the AI intervention and expert-led comparator, outcome measures, and main findings. Discrepancies in data extraction were resolved through consensus with a third reviewer.

### Outcome definitions

The primary outcomes were defined based on objective performance metrics and standardized assessment tools. The primary performance outcome was the interaction effect of feedback on surgical improvement over time, as measured by the ICEMS Expertise Score. This score represents the mean of expert predictions (ranging from −1.00 for novice to 1.00 for expert) computed every 0.2 s of a procedure by a deep learning algorithm, using performance metrics from simulator data [9]. The secondary outcome was learning and skill retention, evaluated by performance on a realistic surgical task (e.g., tumor resection) assessed by both the ICEMS and a blinded Objective Structured Assessment of Technical Skills (OSATS) evaluation. The OSATS rubric comprises six performance categories, each rated on a 7-point Likert scale [24]. Cognitive load was measured via questionnaires using the Mental Effort Scale (MES) on a 7-point Likert scale and the Cognitive Load Index (CLI) on a 5-point Likert scale [25].

### Quality assessment and risk of bias

The risk of bias in randomized controlled trials included in the meta-analysis was evaluated using the Cochrane Risk of Bias 2 (RoB 2) tool [26]. The risk of bias for included observational studies in the narrative synthesis was evaluated using the Newcastle–Ottawa Scale (NOS) [27].

Each study was assessed across several domains, with judgments categorized as 'Low risk,' 'Some concerns,' or 'High risk' of bias. Discrepancies in data extraction or quality assessment were resolved through consensus with a third reviewer.

### Certainty of evidence assessment

The certainty of evidence for each outcome was assessed using the GRADE (Grading of Recommendations Assessment, Development and Evaluation) approach via GRADEpro GDT software [28, 29]. We evaluated the evidence based on five domains: risk of bias, inconsistency, indirectness, imprecision, and publication bias. The overall certainty was categorized as High, Moderate, Low, or Very Low. We started with 'high certainty' for randomized controlled trials and downgraded the rating by one or two levels for serious or very serious limitations in these domains.

### Strategy for data synthesis

A narrative synthesis of the findings from all included studies was conducted to describe the landscape of AI in surgical education. This refers to a structured narrative summary of quantitative study results as recommended by PRISMA 2020 [22]. For the quantitative analysis, a meta-analysis was performed for outcomes reported by at least two studies with sufficient homogeneity. We planned to calculate mean differences (MD) for continuous outcomes (e.g., OSATS scores, ICEMS scores), using a random-effects model to account for anticipated heterogeneity. Statistical heterogeneity was to be assessed using the Chi-square test (Cochrane's Q) and quantified with the I2 statistic. An I2 value greater than 50% was considered indicative of substantial heterogeneity. Our original plan included assessing publication bias if the number of included trials exceeded 10; however, due to the incomplete number of studies, we were unable to perform this assessment. All statistical analyses were planned to be conducted using Review Manager 5.3 software. A *p*-value of < 0.05 was set as the threshold for statistical significance. Leave-one-out sensitivity analyses were performed to characterise the contribution of each integration model and each trial to the pooled estimates.

## Results

### Study selection

**Fig. 1:**
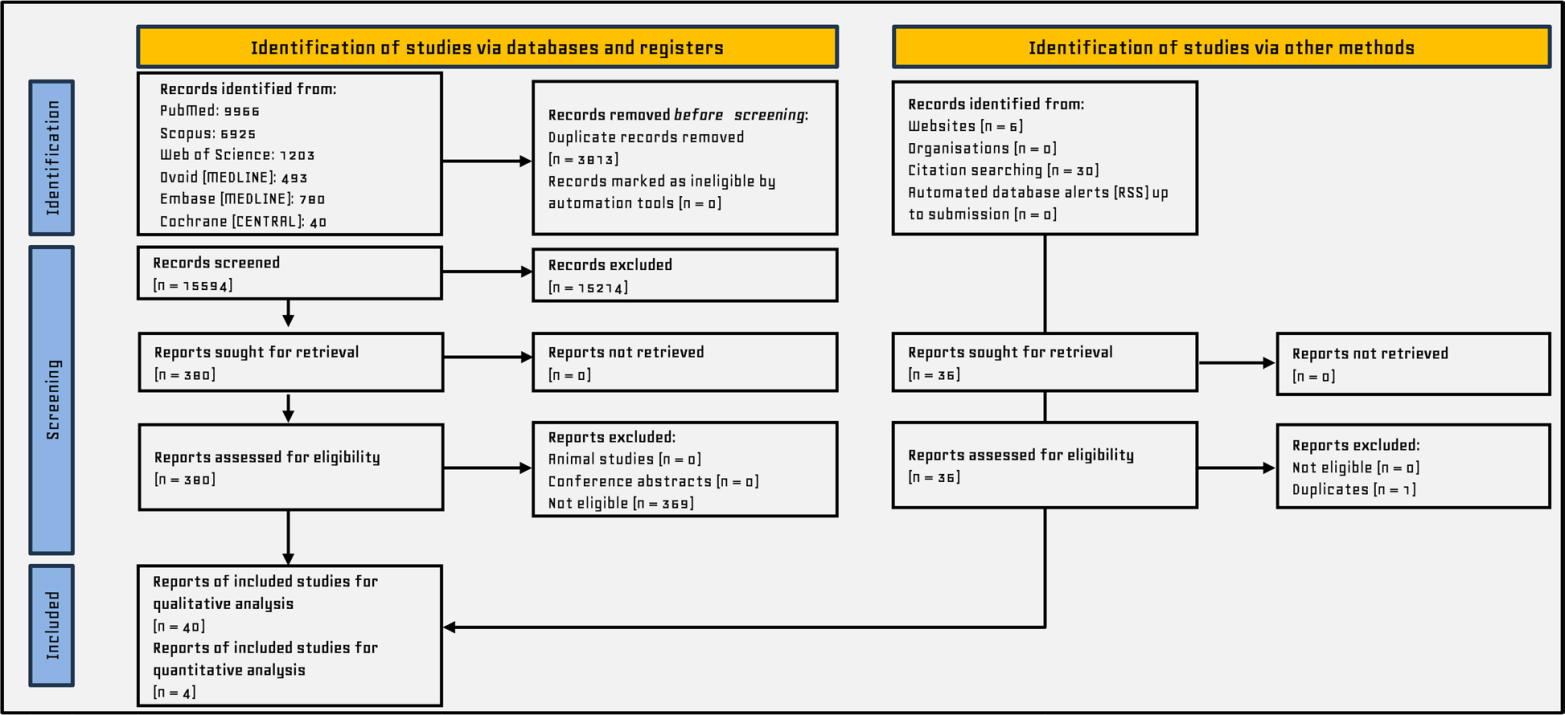
A PRISMA flow chart of our search through different databases

The initial systematic search across PubMed, Scopus, Web of Science, Embase, and Ovid databases yielded 19,367 records. An additional 36 studies were identified through supplementary methods, including citation analysis (*n* = 30) and manual searching (*n* = 6). After the removal of 3,773 duplicate records, a total of 15,594 unique records were advanced to the screening stage. During the title and abstract screening, 15,214 records were excluded as they did not meet the review's eligibility criteria. This left 380 full-text articles that were assessed for eligibility. A thorough full-text review resulted in the exclusion of 369 articles for specific reasons: wrong study design (*n* = 102), wrong intervention (*n* = 66), no comparator group (*n* = 40), wrong population (*n* = 30), wrong outcomes (*n* = 15), mismatched study objective (*n* = 12), and wrong comparison (*n* = 3). Ultimately, this selection process resulted in 40 studies being included in the narrative synthesis. Of these, only four studies met the stringent criteria for inclusion in the meta-analysis. The remaining 36 studies were excluded from quantitative synthesis for the following specific reasons: 25 were validation or development studies of AI assessment tools that lacked a direct comparative instructional arm (i.e., AI tutoring vs. expert instruction); 8 were descriptive reviews, commentaries, or methodological papers without original comparative data; and 4 studies, while comparative, used bespoke or non-standardized outcome measures (e.g., proprietary system scores, task completion times without standard deviation) that could not be statistically pooled with the outcomes (ICEMS, OSATS) reported in the included trials. This heterogeneity in study design and outcome reporting highlights a significant challenge in the field and underscores why our meta-analysis is focused on a specific, comparable subset of the available literature. The complete study selection process is detailed in the PRISMA flow diagram (Fig. 1).

### Narrative synthesis: landscape of AI in surgical education

The 36 studies included in the narrative synthesis represent a broad spectrum of research conducted between 2009 and 2025, originating from various countries, including the USA, Canada, China, Japan, and several European nations, reflecting a global research interest in AI for surgical education. Most studies (*n* = 36) were validation, development, or retrospective/prospective observational studies focused on developing and testing AI tools. Only three studies were RCTs comparing AI-assisted coaching to control methods [30–64] (Table 1).

**Table 1:** Narrative review 1

Study	Title	Publication Year	Country of origin, Location	Study period	Study design
Khalil et al. [30]	Improving microsurgical suture training with automated phase recognition and skill assessment via deep learning	2025	School Of Computing, University of Eastern Finland, Joensuu, Finland	between 2024 and 2025	Validation study
Gustavo et al. [31]	Development of a Laparoscopic Box Trainer Based on Open Source Hardware and Artificial Intelligence for Objective Assessment of Surgical Psychomotor Skills	2018	México	N/A	experimental and comparative methodology
Belmar et al. [32]	Artificial intelligence in laparoscopic simulation: a promising future for large-scale automated evaluations	2022	Santiago, Chile (Catholic University of Chile), with collaborations in the USA	between 2019 and 2022	Validation study
Bodenstedt et al. [33]	Prediction of laparoscopic procedure duration using unlabeled, multimodal sensor data	2019	Germany	NA	experimental and comparative evaluation
Boza et al. [34]	Simulation-trained junior residents perform better than general surgeons on advanced laparoscopic cases	2016	Chile	between 2015 and 2016	experimental study
Chen et al. [35]	Instructive video retrieval for surgical skill coaching using attribute learning	N/A	United States	N/A	Validation study
Cho et al. [36]	Enhancement of Gesture Recognition for Contactless Interface Using a Personalized Classifier in the Operating Room	2018	Korea	N/A	Validation study
Endo et al. [37]	Impact of the Al system on recognition for anatomical landmarks related to reducing bile duct injury during laparoscopic cholecystectomy	2023	Japan	3 years	validation study
Ershad et al. [38]	Automatic Surgical Skill Rating Using Stylistic Behavior Components	2018	USA	N/A	validation study
Fard et al. [39]	Automated robot-assisted surgical skill evaluation: Predictive analytics approach	2018	USA	N/A	validation study
Forestier et al. [40]	Surgical motion analysis using discriminative interpretable patterns	2018	N/A	between 2017 and 2018	validation study
Frischknecht et al. [41]	The Objective Assessment of Experts' and Novices' Suturing Skills Using An Image Analysis Program	2013	N/A	between 2009 and 2013	validation study
Gray et al. [42]	High-Fidelity, Low-Cost, Automated Method to Assess Laparoscopic Skills Objectively	2012	United States	N/A	validation study
Hashimoto et al. [43]	Computer Vision Analysis of Intraoperative Video: Automated Recognition of Operative Steps in Laparoscopic Sleeve Gastrectomy	2019	United States	between 2015 and 2019	retrospective analytical design
Shangdi et al. [44]	Impact of an AI-based laparoscopic cholecystectomy coaching program on the surgical performance: a randomized controlled trial	2024	N/A	16 months	RCT
Kook Jun et al. [45]	Robotic Minimally Invasive Surgical Skill Assessment based on Automated Video-Analysis Motion Studies	2012	N/A	NA	comparative experimental study
Korndorffer et al. [46]	Situating Artificial Intelligence in Surgery: A Focus on Disease Severity	2020	United States	from 2011 to 2019	retrospective analysis
Kumar et al. [47]	Assessing system operation skills in robotic surgery trainees	2012	USA	N/A	longitudinal study
Lavanchy et al. [48]	Automation of surgical skill assessment using a three-stage machine learning algorithm	2021	Switzerland and Germany	between 2014 and 2019	retrospective study
Lee et al. [49]	Evaluation of Surgical Skills during Robotic Surgery by Deep Learning-Based Multiple Surgical Instrument Tracking in Training and Actual Operations	2021	South Korea	NA	retrospective study
Madani et al. [50]	Artificial Intelligence for Intraoperative Guidance: Using Semantic Segmentation to Identify Surgical Anatomy During Laparoscopic Cholecystectomy	2022	37 countries, mainly in the US, France, Canada, the UK, and India	Videos recorded between 2008 and 2019	validation study
Mascagni et al. [51]	A Computer Vision Platform to Automatically Locate Critical Events in Surgical Videos Documenting Safety in Laparoscopic Cholecystectomy Events in Surgical Videos	2021	France	NA	validation study
Monserrat et al. [52]	Automatic supervision of gestures to guide novice surgeons during training	2013	spain	NA	pilot study
Niitsu et al. [53]	Using the Objective Structured Assessment of Technical Skills (OSATS) global rating scale to evaluate the skills of the surgical trainees in the operating room	2012	Japan	between Jan 2007 and Dec 2010	validation study
Okumura et al. [54]	An artificial intelligence-supported system of surgical anatomy recognition may facilitate the understanding of gastrointestinal surgery for medical students	2025	Japan	between Sep 2024 and Mar 2025	Prospective study
Peters et al. [55]	acquisition of surgical suturing techniques Compared to an e-learning and tutor-led course: a randomized controlled trial	2023	Germany	from March to November 2022	multi-arm RCT
Riojas et al. [56]	Knowledge elicitation for performance assessment in a computerized surgical training system	2011	United States	NA	System development and validation study
Rittenhouse et al. [57]	Design and validation of an assessment tool for open surgical procedures	2013	Canada	NA	Validation study
Moglia et al. [58]	Distribution of innate ability for surgery amongst medical students assessed by an advanced virtual reality surgical simulator	2014	Italy	N/A	Prospective cohort study
Sgouros et al. [59]	An automated skills assessment framework for laparoscopic training tasks	2018	Greece	N/A	Prospective observational study
Smith et al. [60]	Deep neural networks are effective tools for assessing performance during surgical training	2021	USA	one year	Retrospective study
Wagner et al. [61]	A learning robot for cognitive camera control in minimally invasive surgery	2021	Germany	N/A	Development and experimental evaluation
Wang et al. [62]	Deep learning with a convolutional neural network for objective skill evaluation in robot-assisted surgery	2018	USA	N/A	validation study
Ward et al. [63]	Surgical data science and artificial intelligence for surgical education	2021	multi-country	N/A	Review Article
Wu et al. [44]	Impact of an AI-based laparoscopic cholecystectomy coaching program on the surgical performance: a randomized controlled trial	2024	China	May 2022 to August 2023	RCT
Zhao et al. [64]	A Machine Learning Approach to Predicting Case Duration forRobot-AssistedSurgery	2019	USA	January 2014 to June 2017	Retrospective study

### Participant and study characteristics

The primary aims of the included studies were generally to develop, validate, or evaluate AI-based systems for surgical training and assessment. The secondary aim was to create intelligent learning and mentoring systems to provide real-time or post-hoc feedback to trainees. The study populations consisted primarily of surgical trainees at various levels — from medical students to residents — and experienced surgeons, including senior residents, fellows, and attending surgeons. Sample sizes ranged from small cohorts of 6–18 individuals to broader populations spanning 8–369 participants. Some studies examined large mixed-institutional cohorts of up to 5,000 healthcare professionals. Video-based datasets ranged from 20 to 54 segments to databases containing up to 1,051 laparoscopic cholecystectomy recordings.

### AI technologies identified

Narrative synthesis highlights a rapidly evolving integration of diverse AI technologies, particularly deep learning models, within general surgery. Deep learning architectures such as Long-Range Recurrent Convolutional Networks (LRCNs) and combinations of Residual Neural Networks (ResNet) with Long Short-Term Memory (LSTM) networks were employed to automate surgical phase recognition and identify operative steps with high accuracy [30, 43]. Traditional machine learning algorithms — including Support Vector Machines (SVM), Random Forest (RF), k-nearest neighbors (k-NN), and Logistic Regression — were primarily applied for objective skill classification based on kinematic data from robotic systems [52–54]. Computer vision techniques, including YOLOv3 and deep semantic segmentation models, were used for real-time identification of critical anatomical landmarks during procedures such as laparoscopic cholecystectomy [37, 50]. Artificial Neural Networks (ANN) were implemented in low-cost simulation platforms to classify trainee expertise, while integrated AI-assisted coaching systems (e.g., SmartCoach and SurgSmart) provided automated feedback and surgical reports [30, 32] (Table 2).

**Table 2:** Narrative review 2

Study	**N**	**Population type**	**AI technology used**	**Description of the used technology**	**Aim of the study**
Khalil et al. [30]	11	surgeons	Deep learning, specifically three variants of modified Long-Range Recurrent Convolutional Networks (LRCNs)	The T-LRCN architecture is designed to model spatial and temporal dependencies jointly. It first performs spatial feature extraction using multiple Time Distributed convolutional layers (which apply the same CNN methods independently to each video frame). These spatial features are then passed to an LSTM layer to extract temporal dependencies, enabling the recognition of subtle sub-phase transitions. The input data consisted of 5-s video clips. A "skipping window" strategy was used to select representative frames and reduce redundancy within the sequence. Data augmentation techniques (horizontal/vertical flips, Gaussian blurring, rotations) were applied to address class imbalance	Create a deep-learning approach for automating phase recognition and skill assessment in microsurgical suturing
Gustavo et al. [31]	20	surgeons and medical trainees	An Artificial Neural Network (ANN)	The system is a physical box trainer combined with open-source technology (a hybrid trainer). The ANN was implemented in the multiplatform Python programming language and executed on a Raspberry Pi board. The input to the ANN consisted of binary images of the traces performed during the pattern cutting task. The selected ANN parameters included an input layer with 2304 neurons (48 × 48 pixel image), a hidden layer with 25 neurons, and an output layer with 2 neurons (experienced or non-experienced). The system uses computer vision (OpenCV library) and augmented reality	Development and objective assessment of a low-cost laparoscopic box trainer using advanced computational technologies
Belmar et al. [32]	less than 500 sample videos for each exercise −369 trainees	trainees participating in a remote laparoscopic simulation program	Artificial intelligence (AI), specifically incorporating machine learning and deep learning methodologies	The AI algorithm analyzes video frames to detect and track the grasper clamps (surgical tools), object movement, and if objects have fallen. It provides a final pass/fail output based on time and object control	Primary Goal: To analyze the agreement between this AI algorithm and expert evaluators in assessing basic laparoscopic-simulated training exercises
Bodenstedt et al. [33]	80	human	Convolutional Neural Networks (CNNs), specifically recurrent CNNs combined with a Gated Recurrent Unit (GRU)	A context-aware method that analyzes the workflow of an intervention online and automatically predicts the remaining duration. The technology uses unlabeled data streams from the endoscopic video (visual channel) and surgical devices (e.g., insufflator, lights). The networks predict procedure progress (a number between 0 and 1), which is then used to compute the remaining procedure duration	The primary aim of the study is to propose and evaluate context-aware methodologies for continuously predicting the duration of laparoscopic surgical interventions
Boza et al. [34]	27	surgical practitioners	simulation training models for teaching and practice, and a motion tracking device for objective performance assessment	Simulation Training: A structured, 16-session advanced laparoscopic training program involving a hand-sewn jejuno-jejunostomy (JJO) in an ex vivo bench model using bovine small bowel. Assessment Technology: The Imperial College Surgical Assessment Device (ICSAD), a motion tracking system, was used to measure the total path length (TPL) traveled by both hands	To assess junior residents trained with simulation performing an advanced laparoscopic procedure in the OR, and compare results to those of general surgeons without simulation training and expert laparoscopic surgeons
Chen et al. [35]	10	resident surgeons	Attribute Learning (to bridge motion measurement and skill understanding), Instructive Video Retrieval, Hidden Markov Model (HMM), and Random Forest (RF)	A vision-based skill coaching system that segments video into primitive actions, rates action units using 6 semantic attributes (e.g., Time and motion, Bimanual dexterity, Depth perception), and uses relative attribute learning to calculate skill ranking. It then retrieves a skill-superior, weakness-specific, and content-similar instructional video clip as automated feedback	The aim of the study is to present a video-based surgical skill coaching system for simulation-based surgical training
Cho et al. [36]	5000	surgeons, physicians, and nurses	Personalized Classifier (a form of Machine Learning)	The system uses a Leap Motion™ sensor to capture hand and finger movements. An automated, personalized classifier, based on seven defined features from the movements, translates these gestures into non-contact commands for computer applications like Picture Archive and Communication System (PACS) and Electronic Medical Record (EMR) in the OR	enhancing technology for use in sterile medical environments, specifically the Operating Room
Endo et al. [37]	230	Videos of Laparoscopic Cholecystectomy	YOLOv3 (You Only Look Once v3) is an object detection algorithm dependent on deep learning	An intraoperative AI system that detects and superimposes landmarks in real-time (30 FPS). It recognizes the extrahepatic bile duct (EHBD), cystic duct (CD), inferior border of liver S4 (S4), and Rouviere sulcus (RS). The display uses transparency based on the AI's confidence level to show ambiguity between landmarks	to evaluate how the Artificial Intelligence (AI) system affects anatomical landmark identification related to reducing bile duct injury during laparoscopic cholecystectomy
Ershad et al. [38]	14	different skill levels in surgery	Regression learner models	Two separate regression learner models were trained for each of the 6 domains of the Global Evaluative Assessment of Robotic Skills (GEARS). One model used sensor measurements (limb kinematics and joint positions), and the other used crowd ratings based on a stylistic behavior lexicon	Evaluate the ability of a proposed stylistic behavior lexicon to automatically rate robotic surgical skill
Fard et al. [39]	8	surgeons	Machine Learning: k-nearest neighbours (k-NN), Logistic Regression (LR), and Support Vector Machine (SVM)	The classifiers use 23 Global Movement Features (GMFs), extracted from the kinematic movement trajectory data captured by the da Vinci surgical robot arms, to quantify surgical skill	develop a predictive method for objective surgical skill assessment based on the movement trajectory of the surgical robot arms
Forestier et al. [40]	8	individuals performing robot-assisted surgical tasks	Machine learning/Pattern Discovery method based on Symbolic Aggregate approximation (SAX) and Vector Space Model (VSM)	The approach uses SAX to discretize continuous kinematic data (multi-dimensional time-series) into a sequence of letters (strings). A sliding window then creates a "bag of words" from these strings. Vector Space Model (VSM) with tf-idf weighting is applied to find and rank discriminative patterns. Classification is performed using Cosine similarity	The primary aim of the study is to identify discriminative and interpretable patterns of surgical practice from recordings of surgical motions
Frischknecht et al. [41]	20	Experts and Novices in Surgical Experience	Image Analysis Program/Simple Computer Algorithm	A computer algorithm used to extract objective variables from digital images of suturing end products. Variables included stitch length, total bite size, number of stitches, and symmetry across the incision ratio	The primary purpose was to objectively assess suturing performance by extracting quantitative data, in an automated fashion, from a summative assessment of suture end-product quality
Gray et al. [42]	15	surgical trainees	Computer vision algorithm. Specifically, the Lucas-Kanade optical flow algorithm	A custom software algorithm applied to digital video data. Two "off-the-shelf" video cameras captured hand and instrument movement from different angles, allowing for 3-dimensional computation of motion. The optical flow algorithm then analyzed the incoming pixels/image frames (voxels) to objectively measure movement smoothness (motion analysis)	The study aimed to prove that computer vision analysis of surgical movements provides an objective basis for technical expertise-level analysis with construct validity
Hashimoto et al. [43]	88	Intraoperative video recordings of laparoscopic sleeve gastrectomy	Deep Learning (SleeveNet), combining a Residual Neural Network (ResNet18) and Long Short-Term Memory (LSTM) neural networks	SleeveNet is the combined model. The ResNet18 (visual model) was trained to extract visual features and identify operative steps from video frames. The LSTM (temporal model) incorporated sequential data ("memory") to augment step identification, improving accuracy	develop and assess Artificial Intelligence (AI) algorithms to identify operative steps in laparoscopic sleeve gastrectomy (LSG)
Shangdi et al. [44]	18	surgeons	SmartCoach (an AI-assisted coaching program)	An AI-assisted coaching program that utilizes an intelligent visualization system to generate a surgical report and pinpoint problems in the procedure. This system enables efficient debriefing and provides AI-enhanced feedback to the coachees	develop and assess an Artificial Intelligence-assisted coaching program for Laparoscopic Cholecystectomy
Kook Jun et al. [45]	6	surgeons with varied levels of expertise	Automated Kinematic Video-Analysis/Statistical Analysis of Micromotions	The system uses automated video processing to segment a primary task into sub-tasks (micromotions) and then applies statistical analysis to evaluate these motions (e.g., economy, repeatability, dexterity). This method adapts principles from well-established motion studies methodologies (like Therbligs) for surgical evaluation	Develop and validate an objective framework for assessing surgical expertise in the Robotic Minimally Invasive Surgical (rMIS) realm, overcoming the limitations of subjective and semi-quantitative metrics currently in use
Korndorffer et al. [46]	1051 video	laparoscopic cholecystectomy video database	Artificial intelligence (AI)	The AI annotated the videos for three main areas: disease severity (using the Parkland Scale), achievement of the Critical View of Safety (CVS) (using the Strasberg Criteria), and intraoperative events	Application validation of Artificial Intelligence AI in surgical quality assurance:
Kumar et al. [47]	8	-surgical trainees and surgical experts	Support Vector Machines (SVM) for supervised classification	The SVM used a polynomial kernel function and an optimization algorithm to find a hyperplane that separates the two classes (expert vs. novice). It was trained on fixed-size, high-dimension feature vectors extracted from the da Vinci system's API motion data (e.g., Cartesian position and velocity) during short-duration system operation events like master clutch activation and camera motion	tracking robotic surgery and trainees to proficiency
Lavanchy et al. [48]	242	video recordings of real-world clinical surgical procedures	Convolutional Neural Network (CNN) (specifically Resnet50-FPN) and Linear Regression Model	A three-stage method: (1) CNN detected and localized surgical instruments (clipper/grasper) in video frames. (2) Motion features (e.g., distance, speed, range of movement) were extracted from the instrument trajectories over time. (3) Linear regression used these features to predict surgical skill ratings	The primary aim of this study was to automate surgical skill assessment in laparoscopic cholecystectomy videos using machine learning algorithms
Lee et al. [49]	54	video segments of robotic surgery procedures	Deep Learning (specifically, a Multiple Surgical Instrument Tracking method)	The system uses Deep Learning to track the positions and motions of multiple surgical instruments from video frames, extracting quantitative metrics like the distance traveled by the instruments. These metrics are then correlated with the surgeon's performance score (e.g., using the Objective Structured Assessment of Technical Skills, OSATS)	The primary aim of this study was to propose a system that quantitatively and automatically assesses the surgical skills of a surgeon during robotic surgery by visually tracking surgical instruments using a deep learning method
Madani et al. [50]	290	Surgical videos of real patients undergoing laparoscopic cholecystectomy	Deep learning	A deep convolutional neural network performing pixel-wise semantic segmentation of safe and danger zones and anatomical structures	Train deep learning models to identify anatomical Landmarks as well as safe and dangerous zones of dissection, and to assess their performance compared to expert annotations
Mascagni et al. [51]	155	Full-length surgical videos of real patients undergoing laparoscopic cholecystectomy (performed by 12 surgeons)	Deep learning(Endodigest)	Combines phase recognition + tool detection Applies workflow rules to automatically infer the time of cystic duct division Extracts 2 min before and 30 s after the event to generate concise CVS(critical view of safety) video documentation	to develop a computer vision platform To automatically locate critical events in surgical videos and provide short video clips documenting the critical view of safety (CVS) in laparoscopic Cholecystectomy (LC)
Monserrat et al. [52]	15	medical students	online LCS (oLCS) string-matching algorithm	Virtual laparoscopic simulator	To develop and evaluate a real-time feedback system that supervises novice surgeons during virtual laparoscopic training by analyzing and correcting their gesture sequences
Niitsu et al. [53]	10	surgical trainees	No AI used	Global Rating Scale	to demonstrate the validity and accuracy of the Objective Structured Assessment of Technical Skills (OSATS) for assessing surgical skills in the Operating room (OR) setting
Okumura et al. [54]	12	Fifth-grade medical students	EUREKA	deep-learning–based semantic segmentation AI	To investigate whether AI-supported identification of surgical anatomy using EUREKA™ helps medical students better understand dissection lines during gastrectomy
Peters et al. [55]	150	medical and dental students	No AI tutoring system was used	an e-learning course (monoscopic) and an HMD-based course (stereoscopic, immersive), both self-directed and a tutor-led course with feedback	Compare the effect of an HMD-based (stereoscopic immersive) course vs an e-learning course vs a tutor-led course on the acquisition of surgical suturing skills in novices
Riojas et al. [56]	38	17 non-medical students, 11 medical students without previous laparoscopic surgery training, 5 medical students with some laparoscopic surgery training,4medicalresidentsand 1 expert surgeon	Fuzzy-logic-based inference system (CAST scoring system)	A Mamdani fuzzy-logic system for proficiency assessment processes five kinematic metrics through expert-defined rules to generate a continuous score. This score, calculated using MIN implication and CENTROID defuzzification in MATLAB, is mapped onto four distinct skill levels	Perform the knowledge elicitation process to formulate expert judgment for the assessment of laparoscopic surgical skills
Rittenhouse et al. [57]	16	11 surgical trainees and 5 experts	No AI used	motion tracking system	design and validate a low-cost model for assessing technical skills in open cholecystectomy using motion tracking and a global rating scale
Moglia et al. [58]	125	121 medical students + 4 expert surgeons	da Vinci Skills Simulator	VR simulator software with multiple exercises testing EndoWrist manipulation, camera control, and needle driving. Metrics include overall scores, economy of motion, force applied, and execution time	Assess the distribution of innate manipulative and psychomotor skills among medical students
Sgouros et al. [59]	74	(36 videos from experts and 38 videos from novices	Computer vision-based framework using Manoeuvre Representation Feature Space (MRFS)	Tracks grasper edges via vanishing points from standard box trainer video; extracts 79 features (trajectories, acceleration, distributions)	present a robust computer vision‐based A framework that utilizes the video stream of the standard box trainer camera, in order to track the graspers and create a novel MRFS that will subsequently be used to classify trainees into experts and novices without any a priori knowledge of the task performed
Smith et al. [60]	254	videos of attending surgeons	Deep Neural Networks	retrained DNN models analyze video clips from VR simulator; uses transfer learning + AutoML to classify performance into expert/intermediate/novice using GEARS-based thresholds	Train DNNs to automatically classify surgical simulation performance matching human instructor GEARS scores
Wagner et al. [61]	20	operations by a single surgeon	machine learning	a three-step learning procedure comprising per ception of data, annotation of data, and machine learning	to develop and evaluate the first context-sensitive, self-learning, autonomous camera-guiding robot for minimally invasive surgery
Wang et al. [62]	8	Surgeons	Convolutional Neural Network (CNN)	A 10-layer deep Convolutional Neural Network (CNN) designed to automatically learn features and classify surgical skill levels directly from raw, multivariate time-series kinematic data (e.g., tool-tip position, velocity) from a robotic surgery system, without the need for manual feature engineering	propose and evaluate a deep learning framework for objective, efficient, and online skill assessment in surgical training
Ward et al. [63]	NA	NA	many technologies(Computer vision (CV), Deep learning (CNNs), Semantic segmentation models (e.g., GoNoGoNet, DeepCVS), Automated workflow recognition models, and AI-based decision-support tools)	describes multiple AI approaches	Review major concepts in surgical data science and AI as applied to surgical education
Wu et al. [44]	18	surgeons	SurgSmart AI system(SmartCoach program)	AI analyzes LC videos for surgical phases, CVS scores, disease severity, critical actions, and generates reports, key timelines for coaching feedback	to develop an artificial intelligence (AI)-assisted coaching Program for LC to enhance surgical education and improve performance
Zhao et al. [64]	424	Elective robot-assisted surgery patients	Multiple machine learning models	1) multivariable linear regression, 2) ridge regression, 3) lasso regression, 4) random forest, 5) boosted regression tree, and 6) neural network	develop an accurate predictive model for robotic-assisted surgery (RAS) case duration

### Key findings by surgical approach/domain

Concerning Laparoscopic Techniques, AI models demonstrated significant effectiveness in objective skill classification and safety enhancement [30, 32, 44, 64]. Studies on laparoscopic cholecystectomy showed that deep learning can identify anatomical landmarks and "Go/No-Go" zones to prevent bile duct injuries [37, 50], and AI-driven coaching tools (e.g., SmartCoach) markedly improved performance and safety metrics for novice surgeons compared to conventional training [45].

In Bariatric Surgery, Deep learning accurately identified operative steps in sleeve gastrectomy, and multi-modal sensor data enabled prediction of procedure durations more efficiently than traditional benchmarks [43, 46]. Hashimoto et al., which describes automated phase recognition in sleeve gastrectomy without a direct AI-tutoring vs. expert-instruction comparison, was retained in the narrative synthesis to characterize the broader landscape of AI in surgery, but was excluded from the quantitative analysis [43].

Robotic Surgery Convolutional Neural Networks (CNNs) and Deep Neural Networks (DNNs) achieved accuracy rates of up to 95.4% in classifying surgeon skill levels from brief kinematic data segments [62]. AI overlays enhanced anatomical understanding during complex procedures such as gastrectomy [54], facilitating objective assessment without constant expert oversight [38].

In Microsurgery and Basic Surgical Tasks, AI models such as the T-LRCN achieved high accuracy in phase recognition and skill assessment for microsurgical suturing tasks [30]. While microsurgery is often classified under plastic surgery, the skills assessed in Khalil et al., including suture placement accuracy, phase recognition, and instrument handling, are directly transferable to basic open surgical tasks performed by general surgery trainees. This study was therefore retained in the narrative synthesis for its relevance to fundamental surgical skill training, but is acknowledged as extending beyond the strict scope of general surgery. Automated image analysis systems and infrared tracking devices were validated as low-cost, effective tools for discriminating expert from novice proficiency in open cholecystectomy and suturing [41].

Collectively, these findings suggest that AI-driven tools offer a scalable and reliable alternative to subjective evaluation, with broad potential to standardize surgical training, provide targeted feedback, and enhance procedural safety and quality across the surgical domain [63] (Table 3).

**Table 3:** Narrative review 3

Study	**operation of the study**	**Surgical specialty**	**Inclusion criteria**	**Conclusion**
Khalil et al. [30]	1- dataset generation and standardization from 11 surgeons 2-data preprocessing and optimization to prepare the video data for deep learning 3-Model Architecture and Training 4-Skill Assessment and Evaluation	Microsurgery (specifically microsurgical suture training)	selection and categorization of the participating surgeons, as well as the standardized design of the microsurgical suturing task used for video data collection	The core results demonstrate the T-LRCN model's high effectiveness in automated microsurgical phase recognition and skill assessment, achieving a peak testing accuracy of 83.85% on unseen data and outperforming benchmark models
Gustavo et al. [31]	1-System Development and Setup 2-Participant Recruitment and Experimental Protocol 3-Execution of Training Tasks 4-Objective Assessment and Analysis 5-Subjective Evaluation	Laparoscopic surgery	volunteers were recruited and how they were categorized into the two classes used for training the Artificial Neural Network (ANN)	The overall finding is that the participants tend to improve their learning curve and dexterity with this laparoscopic training system
Belmar et al. [32]	The operation of this study involved four main phases: data acquisition, AI algorithm development and training, testing and automated assessment, and statistical validation against expert evaluators (EE)	Laparoscopic surgery	selecting the video data used to develop and test the Artificial Intelligence (AI) algorithm designed for the automated assessment of basic laparoscopic skills	The main results of the study demonstrate the feasibility and high level of agreement between the newly developed Artificial Intelligence (AI) algorithm and expert evaluators (EE) in automatically assessing basic laparoscopic simulation exercises
Bodenstedt et al. [33]	The operation of the study involved data acquisition, model development using recurrent Convolutional Neural Networks (CNNs), and rigorous evaluation against multiple datasets and baselines	Colorectal, Upper Gastrointestinal and Bariatric, Hepato-Pancreatico-Biliary, and G.eneral Laparoscopic surgeries	-The recordings must be of laparoscopic surgeries of various procedure types—The endoscopic video stream, Data collected from different surgical devices	The study established the VTD-Net method as the most effective approach for online duration prediction in a diverse laparoscopic setting, confirming that combining unlabeled endoscopic video data and surgical device data is superior to relying on either source alone
Boza et al. [34]	1- Training and Preparation Phase 2- Comparative Assessment in the Operating Room 3- Data Measurement and Analysis	General Surgery and Bariatric Surgery	A-Junior Trainees (PGY1 Residents) 1. Novice Status: Must be novice general surgery residents 2. Training Level: Must be in their postgraduate year 1 (PGY1) 3. Simulation Training: Must have completed a validated 16-session advanced laparoscopy simulation training program B- General Surgeons All Status: Must be general surgeons 2. Training Background: Must have graduated from traditional surgical residency programs 3. Simulation Exclusion: Must have no prior laboratory simulation training in their curricula. C-Expert Bariatric Surgeons 1-certification: Must be certified as laparoscopic bariatric surgery experts 2. Expertise Guarantee: Their expertise was guaranteed by the institutional context: the hospital was a designated Center of Excellence in Bariatric Surgery, having completed more than 400 LRYGB surgeries in 2014	The main results of the study demonstrated that simulation-trained junior residents significantly outperformed conventionally trained general surgeons across all objective and subjective metrics when performing an advanced laparoscopic procedure in the Operating Room
Chen et al. [35]	The operation of the video-based surgical skill coaching system is designed to provide automated, specific, and instructional feedback to trainees	Minimally-invasive surgery	-Participants and Training Environment: The video data. Included the routine training performance of resident surgeons, covering a range of skill levels, specifically experts and novices. -Video Content and Task Requirements: Selected videos were required to be full training sessions. Each full session consisted of 12 Peg Transfer cycles	-High Action Segmentation Accuracy: The system successfully decomposed the videos into primitive action units using motion descriptors and a Hidden Markov Model (HMM). The overall segmentation accuracy was high, achieving 93.5% for expert videos and 80.3% for novice videos.—The experiments validate the effectiveness of the proposed idea and the key algorithms developed to deliver a systematic, vision-based solution for automated skill coaching in simulation-based surgical training
Cho et al. [36]	System Setup and Design, Data Collection and Feature Extraction, Self-Trained Classification, and Experimental Evaluation	Not specified	-Must be able to perform and provide data for the five target. -Mtargetscord approximately 500 samples of skeleton features. -Must participate in the data collection necessary for the "personal basis training" (self-training) methodology, meaning the model learned is unique to that user	The main results of the study demonstrated the significant enhancement of gesture recognition accuracy achieved through the implementation of a personalized automated classifier utilizing a self-training methodology for use in the Operating Room (OR)
Endo et al. [37]	implementing		-The cases must have been performed in Oita University Hospital. -• The cases must have had mild inflammation. -• Each test video was 20 s in length	-The study concluded that the AI system provided significant awareness to both beginners and experts and prompted them to identify anatomical landmarks linked to reducing BDI. The AI system was shown to be effective in determining a safe serosal incision in this research
Ershad et al. [38]	experimental setup utilizing a surgical simulator, human subjects of varying skill levels, extensive sensor data acquisition, and both expert and crowd-sourced video ratings	Robotic surgical tasks	-The subjects were included to represent the full spectrum of different skill levels in robotic surgery	-Both the stylistic behavior lexicon and features extracted from user motion can be used for automatic rating of surgical skill. This capability successfully achieves the study's goal of alleviating the need for time and resource-intensive direct observation by a team of experts to rate a trainee’s performance
Fard et al. [39]	-Data Acquisition and Subject Definition -Feature Extraction and Engineering -Surgical Skill Classification -Performance Evaluation and Validation	Robotic Surgery/Computer-Assisted Surgery	-eight surgeons with varying experience in robotic surgery. -Surgeons were divided into two skill levels, experts and novices	-The study's overall results validate the ability of machine learning techniques, particularly when applied to comprehensive global movement features derived from robotic kinematic data, to provide an objective, repeatable, and accurate automated assessment of surgical skill
Forestier et al. [40]	-Data Acquisition and Preprocessing -Training and Pattern Discovery -Classification and Evaluation -Interpretable Feedback	Robotic Surgery (Minimally Invasive Surgery) and Microsurgery	-Individuals categorized as Novice, Intermediate, or Expert in surgical skills	-The results confirm that the SAX-VSM approach provides a powerful and explanatory method for surgical motion analysis, achieving high accuracy comparable to or exceeding existing 'black-box' methods while uniquely providing actionable, visual feedback for trainee assessment
Frischknecht et al. [41]	structured process of participant performance, digital image capture, objective analysis via a specialized computer algorithm, and statistical comparison	surgical suturing	Novices: They were third-year medical students. They were beginning their surgical clerkship, and they had minimal suturing experience before receiving the specific training for the clerkship. Experts: They were the surgical faculty and residents	The main result of the study is the successful validation of a semiautomated image analysis system that objectively assesses the quality of a completed running suture by demonstrating its ability to significantly discriminate between expert and novice skill levels
Gray et al. [42]	structured process of participant recruitment, standardized task performance, digital data capture using low-cost technology, and automated computer vision analysis	General Surgery	-Beginner Level: PGY-1 general surgery residents. – Advanced Level: PGY-5 general surgery residents or surgical fellows	The level of the trainee became more advanced, the hand and instrument movements became less variable, more efficient, and shorter in duration
Hashimoto et al. [43]	structured process of video selection, human annotation to establish ground truth, data preprocessing, and the training and testing of custom deep neural networks to automatically recognize the surgical steps of laparoscopic sleeve gastrectomy (LSG)	Bariatric Surgery/Minimally Invasive Surgery	-The case had to be a laparoscopic sleeve gastrectomy -The patient's age must have been greater than 18 years -The videos were eligible if they were recorded	The findings indicate that deep learning can be utilized to automatically identify steps of LSG from operative video with a high degree of accuracy
Shangdi et al. [44]	to test the efficacy of an AI-assisted coaching program named SmartCoach on the performance of novice surgeons performing laparoscopic cholecystectomy	General Surgery	They must have been assessed by hepatobiliary experts and deemed to meet the SmartHelp level according to the Zwisch scale	The results of the randomized controlled trial demonstrated that the AI-assisted coaching program significantly improved the performance and safety metrics for novice surgeons performing, compared to traditional self-learning methods
Kook Jun et al. [45]	Comparative experiment utilizing motion studies on a simulated robotic platform to validate the automated assessment framework	Robotic Minimally Invasive Surgery	-The study recruited six subjects with varied levels of expertise. -The subjects were categorized into three groups: experts, intermediates, and novices	the recognition performance improved beyond 70%
Korndorffer et al. [46]	laparoscopic cholecyThectomy	General Surgery	-The videos included in the database were laparoscopic cholecystectomies	-A key finding was that AI annotation and segmentation allowed for extremely efficient video review. -Surgeons were able to review approximately 50 videos per hour based on the AI annotation and segmentation
Kumar et al. [47]	-systematic, longitudinal, and multi-center data collection effort coupled with advanced statistical modeling to objectively assess specific human–machine interface skills in robotic surgery trainees	Robotic surgery training, applicable to specialties including Urology, Cardiothoracic surgery, and Gynecology	-Must be robotic surgery residents and fellows -Trainees had varying amounts of surgical training, but typically had no prior robotic surgery experience. -trainees were characterized by Objective Structured Assessment of Technical Skill (OSATS) scores less than 10	- Once competency in system operation is verified, the trainee can graduate to more complex training tasks that emphasize the skills in which they are still deficient
Lavanchy et al. [48]	automate surgical skill assessment based on the surgeon's instrument and language	Visceral Surgery/General Surgery (implied by laparoscopic cholecystectomy)	-The study screened the institutional video archive exclusively for video recordings of laparoscopic cholecystectomies	-The study concluded that the proposed three-stage machine learning algorithm is effective in distinguishing good and poor surgical skills with high accuracy (87%)
Lee et al. [49]	utilizing deep learning and machine learning models to track surgical instruments and automatically assess surgical skills during robotic surgery	Endocrine Surgery and Robotic Surgery Training	-Videos must be of robotic thyroid surgery utilizing the Bilateral Axillo-Breast Approach. -The videos analyzed must cover the portion of the surgery from the beginning to the locating of the recurrent laryngeal nerve during the thyroid procedure. -The data set included two population types to assess surgical skill across different experience levels	-The study concluded that the proposed deep learning-based system is an effective method to track instruments during robotic surgery, suggesting that this automatic and quantitative evaluation method can replace the current method of surgical skill assessment by surgeons
Madani et al. [50]	Videos were sourced from multiple international datasets, with ten frames extracted per video during the critical view phase. Expert surgeons annotated Go/No-Go zones and key anatomy, and models were trained using tenfold cross-validation. Performance was assessed using IOU, Dice, accuracy, and sensitivity/specificity	laparoscopic cholecystectomy	Laparoscopic cholecystectomy videos, which were performed using the standard bottom-up approach	This study suggests that deep learning can be used to identify the safe and dangerous zones of dissection and other anatomical structures in the surgical field during laparoscopic cholecystectomy with a high degree of performance and the automated computer vision tasks have the potential to augment performance and even, ultimately, be used for real-time decision-support and other quality-improvement initiatives in the future
Mascagni et al. [51]	Decision-support	Laparoscopic Cholecystectomy	full-length lap Chole with consent and CVS manually annotated	A total of 155 LC videos were analyzed: 55 of these videos were used to develop EndoDigest, whereas the remaining 100 were used to test it The time of the Usedtic duct division was automatically located with an MAE of 62.8 130.4s (1.95% of full-length video duration). CVS was assess able in 91% of the 2.5-min-long video clips automatically extracted from The considered test procedures
Monserrat et al. [52]	Expert gesture sequences from 10 surgeons were recorded, and a reference sequence was selected as the closest to all expert strings. Fifteen participants completed four repetitions of two laparoscopic tasks, and the system delivered real-time feedback by comparing their gesture strings to the expert reference	basic laparoscopic skills	Basic	
Niitsu et al. [53]	human evaluation of the surgical trainees using OSATS	General Surgery	postgraduate surgical trainee year 3–5 Participating as main surgeon or first assistant	The postgraduate of the global ranking sc, participating trainee improved with each year of experience. The median scores of all trainees in postgraduate years 3, 4, and 5 were significantly different (p\0.001 for both the main surgeon and first assistant roles
Okumura et al. [54]	Students received a brief gastrectomy lecture, watched a robot-assisted distal gastrectomy video, then drew predicted dissection lines first without AI and again with EUREKA™ overlays showing connective tissue and pancreas. Expert-defined lines were used to measure deviations with ImageJ, students rated the educational value, and the AI’s accuracy was separately tested on 45 frames from three robotic systems	Gastrointestinal Surgery	5th year medical students who were rotating in GI surgery. with no previous observation of GI, s5th-yearith written informed consent	AI significantly improved students’ accuracy in recognizing optimal dissection lines and enhanced their understanding of anatomy during gastrectomy
Peters et al. [55]	Participants did a baseline MRT and 5-min pretest, then a 40-min theory session plus 60-min practice. Post-training, a 5-min 5-minutee was videoed and 40 min by three blinded60-minutee Optical flow and suture counts were computed automatically, with randomisation and CONSORT procedures applied	surgical suturing	novices undergraduate medical and dental students	The use of HMDs with stereoscopic and immersive video provides advantages in the learning experience and should be preferred over a traditional web application for e-learning. Contrary to expectations, feedback is not necessary for novices to achieve a sufficient level in suturing, based only on the number of surgical sutures performed. Achieving training is a good determinant of competence improvement. Nevertheless, feedback still enhances the learning experience. ThCompetenceutomated assessment as an alternative feedback approach could further improve self-directed learning modalities. As a next step, the data from this studycouldbe used to develop such automated AI-based assessments
Riojas et al. [56]	In a study could a coordination task, performance scores for even-numbered test trials were generated by a fuzzy logic system (CAST) built from expert-defined rules and odd-numbered practice trials. The resulting scores were then compared across experience groups to successfully establish construct validity	Minimally invasive surgery	Subjects’ identification numbers were provided according to the five groups of expertise: non-medical students were assigned identification number within the range of [1000, 1999], medical students with no laparoscopic training [2000, 2999], medical students with laparoscopic training [3000, 3999], residents [4000, 4999] and surgeons [5000, 5999]	This research introduces a novel framework for the objective assessment of minimally invasive surgical skills, utilizing a fuzzy logic system to model expert judgment by integrating theoretical knowledge with trainee kinematic data. A key contribution is the new continuity of movement metric, which enhances skill evaluation across tasks. The adaptable framework is designed for future enhancements to continuously advance surgical training standards
Rittenhouse et al. [57]	Participants performed an open cholecystectomy on a porcine-liver simulator, with performance captured using IR and EM tracking plus dual-perspective video. A blinded expert scored performance using an OSATS-style global rating scale, motion metrics were analyzed after removing extraneous movements, and novices completed a survey	Open Cholecystectomy	Eleven novice surgical trainees (less than 10 OCs and less th. an 20 LCs) and five expert surgeons ([58]	Participants performed 26 VR simulator exercises without instruction videos; performance was measured objectively via scores and motion metrics
Moglia et al. [58]	Participants performed 26 VR simulator exercises without instruction videos; performance was measured objectively via scores and motion metrics	robotic surgery	Medical students recruited by open invitation without previous robotic surgery experience, plus expert surgeons acting as controls	In terms of innate aptitude for manipulative and psychomotor abilities, the present investigation has Documented two subpopulations that fall outside the norm for the group of medical students, documented for the study: (i) a small group (6.6 %) Forth a high level and (ii) a larger cohort (11.6 %) with low level (significantly below the norm) innate aptitude for surgery. Exposure to video games An experience did not appear in Normnfluence performances on the da Vinci Skills Simulator
Sgouros et al. [59]	Trainees performed peg transfer and Daot-tying tasks; videos processed via preprocessing (greyscale, motion detection), line detection (LSD), Hough transform for VPs	Laparoscopic surgery	novices (PGY1 residents) and experts (surgeons with > 100 laparoscopic operations)	Achieved 96% correct classification (task-agnostic) and 98–99% (task-specific) distinguishing experts from novices; outperformed prior video-based method by 13%; AI improves teaching by enabling objective, automated, low-cost skill assessment without extra hardware, providing robust metrics for training feedback
Smith et al. [60]	Videos from Basic Robotics Operating Course scored by instructors via GEARS; clips processed and labeled	Robotic surgery	Attending surgeons completing Basic Robotics Operating Course	DNN achieved 83.1% accuracy (Ring & Rail) and 80.8% (Suture the Sponge) matching human GEARS classifications; AI improves teaching by providing consistent, objective, scalable video-based skill assessment without human evaluator burden
Wagner et al. [61]	A robot, pretrained on 20 expert-annotated surgeries where camera guidance, phase, and step were labeled, learned to map system states to camera movements. This learned model was then successfully validated on two robotic platforms during real surgical procedures	Minimally invasive surgery	NA	The cognitive camera robot improved its performance with experience, laying the foundation for a new generation of cognitive surgical robots that adapt to a surgeon’s needs
Wang et al. [62]	he study used the public JIGSAWS dataset of robotic surgery kinematic data. A CNN model was trained to classify surgeon skill level (Novice, Intermediate, Expert) based on short (1–3 s) windows of raw motion data from tasks like suturing and knot-tying. Performance was validated using cross-validation techniques	Robot-assisted Minimally Invasive Surgery	surgeons with varying robotic surgical experience	The AI (CNN) improved the potential for teaching quality by enabling efficient, online, and objective skill assessment. It could decode skill level from very short (1–3 s) data windows with high accuracy (up to 95.4%), paving the way for real-time feedback without needing an expert to observe an entire procedure or manually analyze complex data
Ward et al. [44]	Surgeons performed 5 supervised LCs each; coaching group got 30-min AI-report-based sessions post-surgery; while self-learning reviewed the datasets	laparos30-minutecholecystectomy	patients criteria are: 1) an age of 18 years or older, 2) a confirmed surgical Indication for patients: 3) no other concomitant procedures during this hospital stay, 4) no severe comorbidities, while the Surgeon must meet SmartHelp level according to (Zwisch sc, ale)	The AI-assisted surgical coaching program effectively improved surgical performance and safety for novice surgeons in LC procedures. The model holds significant promise for advancing surgical education
Zhao et al. [64]	Random sample of cases analyzed; baseline (scheduled duration) vs 6 ML models; best model applied to full dataset for booking accuracy comparison	Elective robotic casesMulti-specialty robot-assisted surgery	Elective robotic cases (confirmed via Common PMulti-specialty (CPT) code S2900 and procedures with ≥ 10 instances	.This study shows that using various machine learning approaches can improve the accuracy of the RS case length predictions, which will increase utilization of this limited resource. Further work is needed to operationalize these findings

### Summary of narrative synthesis findings

Key findings from the narrative synthesis indicate that AI can provide objective, automated, and scalable assessment of surgical skills, often with high accuracy compared to expert human evaluators. AI-assisted coaching programs significantly improved surgeon performance and safety metrics compared to self-learning. Furthermore, AI systems demonstrated the ability to automatically identify surgical phases, assess the critical view of safety, and provide real-time decision support, thereby enhancing both training efficiency and potential patient safety.

Across the 40 included studies, several consistent themes emerged: (a) deep learning architectures (particularly CNNs and LSTMs) were the most commonly employed AI technologies; (b) the majority of studies (n = 25) focused on tool or system validation rather than comparative effectiveness; (c) laparoscopic cholecystectomy was the most frequently studied procedure; (d) sample sizes were generally small; and (e) only three studies were RCTs, highlighting a critical gap in high-quality comparative evidence. Geographic distribution was concentrated in North America and East Asia, with limited representation from LMICs despite the stated global surgical deficit.

### Risk of bias assessment — narrative synthesis

**Fig. 2:**
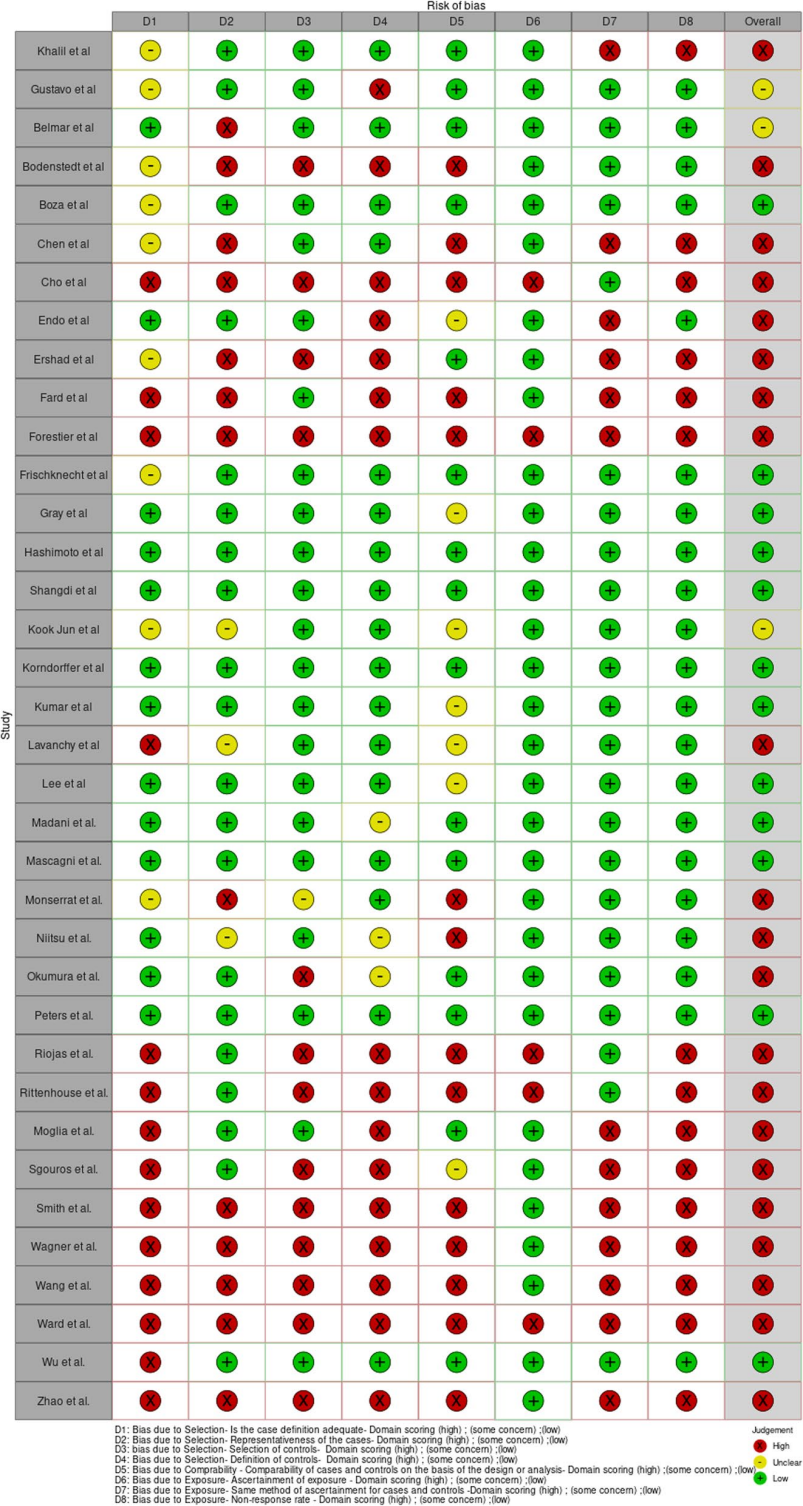
NOS risk of bias assessment

The methodological quality of the 36 studies assessed using the NOS displayed considerable variability. Twenty-one studies (58%) were judged to have a high overall risk of bias, primarily due to deficiencies in selection and comparability domains. Twelve studies exhibited low risk, and three showed an unclear risk. Although outcome assessment was consistently strong across studies, frequent concerns regarding follow-up adequacy compromised the long-term reliability of various findings. The broad findings regarding the development and validation of AI tools in the narrative synthesis should therefore be interpreted with significant caution (Fig. 2).

### Meta-analysis results

#### Study characteristics tables

The four studies detailed in Table 4 were conducted between 2019 and 2025, primarily in Canada and East Asia, highlighting a growing international focus on artificial intelligence in surgical education. Each study utilized simulation methodologies to investigate early technical skill acquisition among novice learners, including medical students and junior medical interns. The methodological quality was notably high, comprising three randomized controlled trials and one prospective pilot study. Participant numbers varied from 70 to 97, and all studies were executed within well-defined, short-term training periods in controlled simulation settings, prioritizing internal validity over direct clinical application. The main aim of these studies was to assess the effectiveness of AI-based tutoring and feedback systems in comparison to traditional expert-led instruction or minimal feedback. The interventions utilized advanced machine learning and deep learning–based AI systems integrated into simulation platforms. Collectively, these studies demonstrate a shift toward AI-enabled, data-driven assessment and coaching in surgical education, with AI systems demonstrating non-inferiority and sometimes superiority over traditional teaching methods in developing technical skills and learner confidence, highlighting their potential as standardized tools in surgical training. The AI systems in the four meta-analyzed trials, while all simulation-based, differed in their pedagogical approach. The system used by Fazlollahi et al. and Yilmaz et al. was an Intelligent Tutoring System integrated with the NeuroVR simulator. It provided real-time, automated feedback based on performance metrics (e.g., instrument path length, tissue damage) compared against an expert model, delivering corrective prompts via on-screen text and audio cues. The system in Giglio et al. was an 'AI-augmented' model where the AI (SurgSmart) generated a post-procedure report with objective metrics and video highlights of critical errors, which was then used by a human expert to deliver personalized feedback. The system in Yang et al. provided feedback on suturing and ligature skills, assessing parameters like knot security and loop formation, and providing a summary score and narrative feedback post-task. These differences in feedback timing (real-time vs. post-hoc) and delivery method likely contribute to the heterogeneity in outcomes [19, 65–67] (Table 4).

**Table 4:** Study characteristics of included studies

Study (Author, Year, Journal)	Study Design & Country	Sample Size & Population	AI Technology Used & Description	Surgical Domain & Setting	Aim of Study	Key Conclusion
Fazlollahi et al (2022) [19] JAMA Network Open	RCT (3-arm) Canada	*N* = 70 Medical students (years 0–2; 4 institutions)	Virtual Operative Assistant (VOA) ML + deep learning; audiovisual feedback on 4 metrics (bleeding, force, separation, acceleration) during VR brain tumor resection simulation	Basic surgical training (VR neurosurgical resection)	Compare VOA AI tutoring vs remote expert instruction vs no feedback on skill acquisition, transfer, and cognitive/affective outcomes in VR simulation	VOA demonstrated superior performance outcomes and skill transfer with equivalent OSATS ratings and cognitive/emotional responses compared to remote expert instruction, supporting its use in scalable simulation training
Giglio et al (2025) [65] JAMA Surgery	3-arm RCT Canada	*N* = 87 Medical students (years 1–2; 4 Quebec schools)	ICEMS (Intelligent Continuous Expertise Monitoring System) Multi-algorithm AI assessing bimanual skills at 0.2-s intervals; real-time action-oriented verbal feedback and composite expertise score	Basic surgical training (VR brain tumor resection)	Investigate effect of AI-augmented human instruction (educator provided with ICEMS quantitative performance data) on technical skill acquisition during simulation	Personalized expert instruction augmented with AI data enhanced performance and skill transfer vs AI tutor alone, emphasising the continued importance of human input in AI-based surgical training
Yang et al (2019) [66] J Educ Eval Health Prof	Prospective pilot study (3-group) Taiwan	*N* = 72 Novice medical interns (no prior suturing training)	WKS-2RII System (Waseda–Kyoto–Kagaku) AI evaluates suturing/ligature on artificial skin; generates quantitative Technical Performance (TP) score from 6 metrics compared to expert standard	General Surgery (suturing and ligature skills)	Improve skill acquisition and confidence of medical interns through a novel expert-led + AI-assisted tutoring program	Adding an expert-led + AI-assisted tutoring course to the standard surgical curriculum demonstrated potential value for improving technical performance and intern confidence in suturing and ligature skills
Yilmaz et al (2024) [67] Scientific Reports	3-arm RCT Canada	*N* = 97 Medical students (years 1–4)	ICEMS (Deep Learning: LSTM networks) Assesses performance at 0.2-s intervals; real-time auditory feedback + post-hoc video error analysis on 5 metrics (bleeding/tissue risk, aspirator/bipolar force, instrument tip separation)	Basic surgical training (VR neurosurgical resection)	Compare efficacy of tailored ICEMS AI feedback vs in-person expert instruction in simulated surgical skills training	Real-time multifaceted ICEMS AI feedback was comparable to in-person expert instruction, offering a scalable, high-fidelity training approach with quantifiable, objective performance metrics

*Abbreviations: AI* Artificial Intelligence, *ICEMS* Intelligent Continuous Expertise Monitoring System, *LSTM* Long Short-Term Memory, *ML* Machine Learning, *OSATS* Objective Structured Assessment of Technical Skills, *RCT* Randomised Controlled Trial, *TP* Technical Performance, *VR* Virtual Reality, *WKS-2RII* Waseda–Kyoto–Kagaku Suture No. 2 Refined II

References: [1] Fazlollahi AM et al. JAMA Netw Open. 2022; 5(2):e2149008 [19]. [2] Giglio B et al. JAMA Surg. 2025; 160(9):993–1003 [65]. [3] Yang YY, Shulruf B. J Educ Eval Health Prof. 2019; 16:7 [66]. [4] Yilmaz R et al. Sci Rep. 2024; 14(1):15,130 [67]

#### Risk of bias assessment

**Fig. 3:**
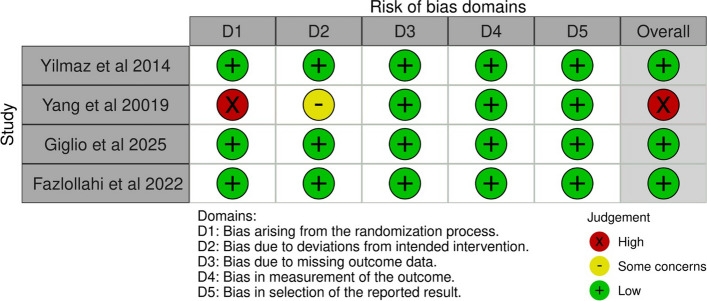
ROB2 risk of bias assessment

The Risk of Bias assessment indicates a predominantly low risk across the included studies, with 75% of trials demonstrating high methodological rigor across all five assessment domains. Yang et al. was the sole outlier, exhibiting a high overall risk primarily due to deficiencies in the randomization process and concerns regarding intervention deviations [66]. The remaining three studies maintained low risk in all categories, providing a high-quality evidence base for AI-driven surgical instruction (Fig. 3).

#### ICEMS expertise score (Table 5: summary of findings)

**Table 5:** Summary of findings — meta-analysis

Outcome (with definition)	No. of Studies	No. of Participants	Pooled Mean Difference (MD) [95% CI]	*p*-value	Heteroge-neity (I^2^)	Certainty of Evidence (GRADE) & Interpretation
PRIMARY OUTCOMES						
ICEMS Expertise Score (Surgical Performance) Mean of expert predictions (− 1.00 novice to + 1.00 expert), computed every 0.2 s by deep learning algorithm from VR simulator metrics	**3**	171	**0.25** **[− 0.25 to 0.76]**	0.33	97% (*p* < 0.00001)	⨁⨁⨁◯ MODERATE High heterogeneity (I^2^ = 97%) primarily driven by Fazlollahi et al. (2022) [19] due to differences in outcome measurement scale Sensitivity analysis (excluding Fazlollahi et al.): I^2^ = 0%, MD 0.00 [− 0.19 to 0.19]; *p* = 1.00 — no significant difference between AI and expert instruction
OSATS Evaluation (Skill Retention) Blinded assessment using OSATS rubric (6 categories, 7-point Likert scale) on realistic surgical task	**3**	159	**0.20** **[0.01 to 0.39]**	0.04	0% (*p* = 1.00)	⨁⨁◯◯ LOW Serious concerns: • Pooled result dominated (79.9% weight) by Yang et al. (2019) [66], assessed as having critical risk of bias • Total sample (*N* = 159) falls below optimal information size (OIS)
COGNITIVE LOAD SUBSCALES (MES 7-point & CLI 5-point Likert scales)						
Germane Cognitive Load	**3**	171	** − 0.05** **[− 0.10 to − 0.01]**	0.02	0% (*p* = 0.90)	⨁⨁⨁◯ MODERATE Result significant (*p* = 0.02) but effect is small; upper CI (− 0.01) very close to null. *N* = 171 below OIS
Intrinsic Cognitive Load	**3**	171	** − 0.07** **[− 0.14 to 0.01]**	0.08	0% (*p* = 0.92)	⨁⨁⨁◯ MODERATE Narrow CI [− 0.14 to 0.01] excludes large effects in either direction. *N* = 171 below OIS
Extraneous Cognitive Load	**3**	171	**0.23** **[0.05 to 0.41]**	0.01	0% (*p* = 0.61)	⨁⨁⨁◯ MODERATE Statistically significant; AI group showed higher extraneous cognitive load. *N* = 171 below OIS

*Abbreviations: CI* Confidence Interval, *CLI* Cognitive Load Inventory, *GRADE* Grading of Recommendations, Assessment, Development and Evaluations, *ICEMS* Intelligent Continuous Expertise Monitoring System, *MD* Mean Difference, *MES* Mental Effort Scale, *OIS* Optimal Information Size, *OSATS* Objective Structured Assessment of Technical Skills, *RCTs* Randomised Controlled Trials

Footnotes: (a) High heterogeneity resolved by sensitivity analysis excluding Fazlollahi et al. (2022) [19] [scale difference]. (b) Total sample *N* = 124 below OIS. (c) Pooled result dominated (79.9%) by Yang et al. (2019) [66] with critical risk of bias. (d) Total sample *N* = 159 below OIS. (e) Significant (*p* = 0.02) but upper CI close to null; *N* = 171 below OIS. (f) CI narrow; *N* = 171 below OIS. (g) *N* = 171 below OIS

**Fig. 4:**
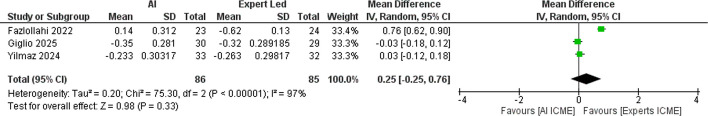
Forest plot ICEMS

The first primary outcome was the ICEMS Expertise Score, a measure of surgical performance. The meta-analysis included three RCTs with a total of 171 participants. The analysis (Fig. 4) revealed no statistically significant difference between AI-tutored and expert-led instruction groups, with a pooled mean difference (MD) of 0.25 (95% CI, −0.25 to 0.76; *p* = 0.33). However, the analysis showed substantial heterogeneity among the studies (I2 = 97%; *p* < 0.00001). This high level of heterogeneity is primarily driven by Fazlollahi et al., who reported a large, statistically significant effect favoring the AI group (MD = 0.76), whereas the other two studies found negligible, non-significant differences. This substantial discrepancy suggests that the effectiveness of AI tutors on machine-measured performance is highly dependent on the specific AI system, the learning task, or other unmeasured study-level factors. A simple pooled estimate would obscure this critical variability [19]. Sensitivity analysis was done removing Fazollahi et al., Heterogeneity was resolved, and no statistically significant results were found (*p*-value = 1) [19].

A leave-one-out sensitivity analysis confirmed Fazlollahi et al. [19] as the sole driver of the observed trend and statistical instability. Omitting Fazlollahi 2022 completely resolved heterogeneity (I2 = 0%) and nullified the effect (MD = − 0.00; 95% CI, − 0.10 to 0.10; *p* = 0.998). Omitting either Giglio 2025 or Yilmaz 2024 left heterogeneity near-intact (I2 = 98.0% and 98.3%, respectively) and results remained non-significant. This pattern confirms that the pooled ICEMS estimate cannot be interpreted as a coherent average effect of AI tutoring across trials, and that the apparent advantage in Fazlollahi et al. is best understood as a single-study finding rather than a generalisable pooled effect. (Supplementary file 3: leave one out analysis).

#### OSATS evaluation

**Fig. 5:**
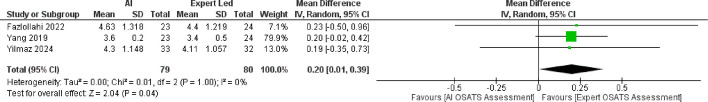
Forest plot OSATS

The second primary outcome was learning and skill retention, as evaluated by OSATS. The meta-analysis included three studies with a total of 159 participants. The pooled results (Fig. 5) showed a statistically significant difference favoring the AI-tutored group, with an MD of 0.20 (95% CI, 0.01 to 0.39; *p* = 0.04). This suggests that AI-based training may lead to slightly better surgical skills as measured by the OSATS rubric. The analysis showed no statistical heterogeneity (I2 = 0%; *p* = 1.00).

However, this pooled estimate was heavily weighted (79.9%) by Yang et al., a study assessed as having high risk of bias due to deficiencies in the randomisation process and concerns regarding intervention deviations [66]. The certainty of this evidence was rated as low (see GRADE assessment in Table 6). A leave-one-out sensitivity analysis was performed to characterise the robustness of this finding. Omission of any single study—Fazlollahi 2022, Yang 2019, or Yilmaz 2024—rendered the estimate non-significant, with p-values ranging from 0.052 to 0.354. The point estimate remained stable at MD = 0.20 across all omission scenarios, but the 95% confidence intervals crossed the null in each case, indicating that the OSATS finding is sensitive to the inclusion of any single trial and should be interpreted with substantial caution.(Supplementary file 3: leave-one-out analysis).

**Table 6:** GRADE evidence profile

Outcome & Participants	CERTAINTY ASSESSMENT						SUMMARY OF FINDINGS				
**Participants** **(Studies)** **Follow-up**	**Risk of Bias**	**Inconsistency**	**Indirectness**	**Imprecision**	**Publication Bias**	**Overall Certainty**	**Event** **Rate** **(Expert)**	**Event** **Rate** **(AI)**	**Relative** **Effect** **(95% CI)**	**Risk with** **Expert** **Instruction**	**Risk** **Difference** **with AI**
ICME Score 124 (3 RCTs)	Not serious	Not serious	Not serious	**Serious**	None	⨁⨁⨁◯ **MODERATE** **(a, b)**	61	63	N\A	61	**MD 0 ICME score** **(0.1 lower to** **0.1 higher)**
OSATS Score 159 (3 RCTs)	Serious	Not serious	Not serious	**Serious**	None	⨁⨁◯◯ **LOW** **(c, d)**	80	79	N\A	80	**MD 0.2 OSATS score** **higher** **(0.01 higher to** **0.39 higher)**
Germane Cognitive Load 171 (3 RCTs)	Not serious	Not serious	Not serious	**Serious**	None	⨁⨁⨁◯ **MODERATE** **(e)**	85	86	N\A	85	**MD 0.05 score** **lower** **(0.1 lower to** **0.01 lower)**
Intrinsic Cognitive Load 171 (3 RCTs)	Not serious	Not serious	Not serious	**Serious**	None	⨁⨁⨁◯ **MODERATE** **(f)**	85	86	N\A	85	**MD 0.07 score** **lower** **(0.14 lower to** **0.01 higher)**
Extraneous Cognitive Load 171 (3 RCTs)	Not serious	Not serious	Not serious	**Serious**	None	⨁⨁⨁◯ **MODERATE** **(g)**	85	86	N\A	85	**MD 0.23 score** **higher** **(0.05 higher to** **0.41 higher)**

*Abbreviations: CI* Confidence Interval, *GRADE* Grading of Recommendations, Assessment, Development and Evaluations, *ICME* Inverse Consistency Mean Error, *MD* Mean Difference, *OSATS* Objective Structured Assessment of Technical Skills, *RCTs* Randomised Controlled Trials

Explanations: (a) Results based on sensitivity analysis excluding Fazlollahi et al. (2022) [19] to resolve heterogeneity arising from outcome measurement scale differences. (b) *N* = 124 below optimal information size (OIS). (c) Pooled result heavily dominated (79.9% weight) by Yang et al. (2019) [66], assessed as having critical risk of bias. (d) *N* = 159 below OIS. (e) Statistically significant (*p* = 0.02), effect small, upper CI (− 0.01) near null; *N* = 171 below OIS. (f) *N* = 171 below OIS; CI narrow and excludes substantial effects in either direction. (g) *N* = 171 below OIS

#### Cognitive load

The analysis for germane cognitive load showed a statistically significant difference between groups (MD = −0.05; 95% CI, −0.10 to −0.01; *p* = 0.02), with no heterogeneity (I2 = 0%; *p* = 0.90). The result indicates that the AI-tutored group experienced a slightly lower germane cognitive load compared to the expert-led instruction group.

For intrinsic cognitive load, the meta-analysis found no statistically significant difference between the AI and expert-led groups (MD = −0.07; 95% CI, −0.14 to 0.01; *p* = 0.08). There was no evidence of heterogeneity (I2 = 0%; *p* = 0.92).

**Fig. 6:**
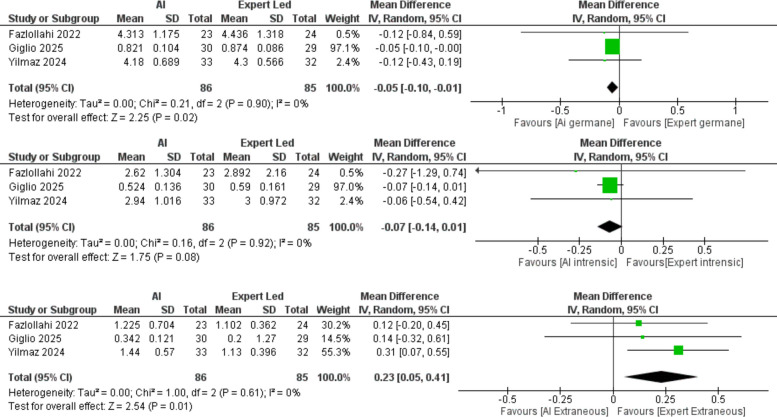
Forest plot cognitive load

The analysis of extraneous cognitive load revealed a statistically significant difference, with the AI-tutored group reporting a higher load than the expert-led group (MD = 0.23; 95% CI, 0.05 to 0.41; *p* = 0.01). This suggests that the design of the AI interventions may have imposed an additional 0.23 units of mental effort, not directly related to the learning task itself. No heterogeneity was detected (I2 = 0%; *p* = 0.61). (Fig. 6).

Leave-one-out sensitivity analyses were performed for each cognitive-load outcome. For germane cognitive load, the pooled result was robust to omission of Fazlollahi 2022 and Yilmaz 2024 but lost significance when Giglio 2025 was omitted (MD = − 0.12; 95% CI, − 0.40 to 0.16; *p* = 0.40), attributable to the substantially wider confidence interval rather than a direction change, the result is directionally consistent but depends on Giglio’s contribution to precision. For intrinsic cognitive load, the null finding was stable across all omission scenarios. For extraneous cognitive load, the result remained significant when Fazlollahi 2022 or Giglio 2025 were omitted, but omitting Yilmaz 2024 rendered the effect non-significant (MD = 0.13; 95% CI, − 0.14 to 0.39; *p* = 0.34), indicating that this finding, while directionally consistent, lacks sufficient robustness across the available evidence base. (Supplementary file 3: leave-one-out analysis).

#### Certainty of evidence assessment

Regarding the ICEMS score, the analysis showed no significant difference between AI tutoring and expert instruction (MD = 0.00; 95% CI [−0.10, 0.10]). The certainty of this evidence was rated as moderate, downgraded only for imprecision due to the relatively small total sample size.

Conversely, regarding global operative performance (OSATS score), AI tutoring demonstrated a statistically significant improvement compared to expert instruction (MD = 0.20 points higher; 95% CI [0.01, 0.39]). However, the certainty of this evidence was rated as low. We downgraded due to imprecision and risk of bias, as the analysis was heavily weighted (79.9%) by Yang et al. study with critical methodological limitations [66].

We found moderate certainty evidence regarding cognitive load outcomes. The analysis indicated that AI tutoring resulted in significantly higher extraneous load (MD = 0.23; 95% CI [0.05, 0.41]), suggesting higher distraction levels compared to expert instruction. Furthermore, the AI group showed slightly lower germane load (MD = −0.05; 95% CI [−0.10, −0.01]). There was no significant difference in intrinsic cognitive load (MD = −0.07; 95% CI [−0.14, 0.01]) (Table 6: GRADE assessment).

## Discussion

This systematic review and meta-analysis is, to our knowledge, the first to quantitatively compare AI-tutored instruction with traditional expert-led teaching for simulated surgical skill acquisition. The integration of AI into surgical education has been identified as a transformative development, with the potential to offer personalized, scalable, and objective training environments through technologies such as virtual and augmented reality simulators [68, 69]. While numerous studies have explored the development and validation of AI tools for skill assessment and feedback [70, 71], their effectiveness as a teaching tool requires review of existing evidence. This need is underscored by a global surgical deficit: 5 billion people lack access to safe surgery, and an estimated 143 million additional procedures are needed annually, primarily in low- and middle-income countries (LMICs) [72]. AI-assisted instruction represents a potentially transformative tool to help address this gap by overcoming geographical barriers and providing standardized education where expert mentors are unavailable [72, 73].

These findings align with a growing body of literature supporting AI integration into surgical training while highlighting current limitations. Numerous studies have demonstrated that AI can provide objective, automated, and scalable assessment of surgical skills, often with high accuracy compared to human evaluators [30, 32, 62]. AI-driven coaching tools have been shown to significantly improve performance and safety metrics in novice surgeons compared to conventional or self-learning methods [44]. Our finding that AI tutoring is at least as effective as expert instruction is consistent with this literature [70, 73, 74]. Notably, Giglio et al. [65] found that AI-augmented personalized expert instruction yielded better performance and skill transfer than an AI tutor alone, supporting our conclusion that the optimal role for AI may not be as a standalone instructor but as a component of a hybrid model.

The absence of a significant difference in ICEMS scores suggests that, from a purely quantitative standpoint based on simulator metrics, AI tutors perform comparably to human tutors. However, the substantial heterogeneity (I2 = 97%) in this outcome, largely driven by Fazlollahi et al. [19], indicates considerable variability across AI systems. Sensitivity analysis removing this study resolved the heterogeneity and confirmed no significant difference, implying that the specific design and algorithms of an AI system may substantially influence its effectiveness. An important conceptual consideration is that AI tutoring systems are not inherently "objective" in an absolute sense; they are trained on performance data labelled by human experts, meaning they operationalize expert standards rather than transcend them. Their primary advantages are scalability, consistency, and granular data-driven analysis, which augment rather than replace human expert judgment.

When performance was evaluated by blinded human experts using the OSATS rubric, the AI-tutored group demonstrated a small but statistically significant superiority (MD 0.20). The clinical and educational significance of this finding, however, warrants careful interpretation. The OSATS rubric comprises six categories rated on a 7-point Likert scale [24]. Standard-setting studies have established that competency thresholds on the OSATS require score differences of several points on the total scale [75]; for example, Szasz et al. established a cut-point of 21.04/35 for technical competence in laparoscopic cholecystectomy. Importantly, no formally validated minimum clinically important difference (MCID) has been established for OSATS scores in novice surgical trainees. Given the scale structure, a mean difference of 0.20 points is unlikely to represent a clinically perceptible improvement in surgical performance. Furthermore, this pooled estimate was heavily weighted (79.9%) by Yang et al. [66], a study with high risk of bias, and the certainty of evidence was rated as low. The observed advantage, while statistically favoring AI, represents a small effect size whose practical relevance in a real-world training curriculum remains uncertain.

The cognitive load findings warrant nuanced interpretation rather than a unidirectional conclusion. Higher extraneous load in the AI group suggests that the design of current AI interfaces may introduce distracting or confusing elements compared to direct human interaction, mental effort unrelated to the learning task itself [65].

The finding of lower germane cognitive load, however, admits multiple interpretations. First, cognitive efficiency: if OSATS scores improved despite lower self-reported germane load, this may reflect more efficient learning, learners constructing effective mental schemas with less perceived effort, consistent with the cognitive efficiency framework in medical education [76, 77]. Second, cognitive resource competition: cognitive load theory posits that intrinsic, extraneous, and germane loads are additive and compete for limited working memory capacity [76]. If extraneous load was elevated in the AI group (e.g., due to unfamiliarity with the AI interface), this may have consumed cognitive bandwidth that would otherwise have been available for germane processing, effectively constraining rather than reflecting reduced learning effort. Third, the self-report instruments used to measure cognitive load (MES and CLI) may not reliably distinguish between reduced effort due to efficient learning versus reduced effort due to cognitive overload from other sources [77]. These interpretations suggest that lower germane cognitive load in the AI group is not necessarily a negative finding. Future AI tutor design must prioritize minimizing extraneous load while investigating whether feedback promotes or replaces the essential cognitive processes underlying schema construction.

Taken together, these results support a hybrid "centaur" model where AI's data-driven, scalable feedback augments the nuanced, adaptive guidance of a human expert [20]. This approach leverages AI's capacity for objective, continuous data analysis while retaining the human expert's ability to contextualize feedback, manage cognitive load, and provide mentorship. The current evidence does not, however, justify replacing human instructors with AI; rather, it points to integration as the more defensible and clinically meaningful direction.

### AI as augmentation, not replacement, of the expert

The central message is that AI tutoring is best viewed as an augmentation of expert-led surgical training, not a replacement. In trials directly comparing AI to expert instruction, AI showed non-inferiority but not consistent superiority on externally validated OSATS scores (Fazlollahi et al., Yilmaz et al.). The trial specifically designed to test augmentation (Giglio et al.) found that an expert using AI-derived data to personalise feedback outperformed both AI alone and an expert reading identical AI-scripted words. In the only hybrid-pathway trial in non-VR conditions (Yang et al.), adding an AI self-assessment system to expert tutoring produced higher OSCE scores, with a dose–response effect across one, two, and three AI sessions. Together, these findings do not support a model in which AI replaces the expert or alters the fundamental structure of surgical pedagogy. The expert’s role—providing context, modelling judgement, contextualising risk, and translating skills to the operating room—remains the anchor of effective training.

### Limitations

Several limitations must be acknowledged. First, the OSATS pooled estimate was heavily weighted (79.9%) by Yang et al., a study with high risk of bias, and the certainty of evidence for this outcome was rated as low. Second, the four included trials operationalise two integration models, AI-substitutive (Fazlollahi et al., Yilmaz et al.) and AI-augmentative (Giglio et al., Yang et al.), which test complementary aspects of the same pedagogical question. This is a deliberate feature of the synthesis under the augmentation-vs-replacement framing of the Aim, but it introduces a degree of clinical heterogeneity into the pooled estimates that readers should consider when interpreting effect sizes; pre-specified leave-one-out sensitivity analyses are reported in the Results. Third, formal inter-rater reliability (e.g., Cohen's kappa) was not calculated for the eight-reviewer screening process, which may limit reproducibility; average kappa values of 0.77–0.86 have been reported as benchmarks for systematic review screening [78]. Fourth, no validated MCID has been established for OSATS scores in novice surgical trainees, making the clinical significance of small score differences inherently uncertain. In the absence of a validated MCID, we contextualized the observed 0.20-point difference against the OSATS scale structure and published competency cut-points rather than relying on informal thresholds that lack empirical support. Fifth, the primary limitation is the small number of studies (four) eligible for quantitative analysis restricts the power of our subgroup and sensitivity analyses. Although our narrative synthesis included 40 studies, the lack of standardized outcome reporting across the field prevented a larger meta-analysis. This highlights a critical need for consensus on core outcome sets for research in surgical education technology. Furthermore, the studies included were conducted in simulated environments. The transferability of skills learned in a virtual environment to the high-stakes reality of the operating room remains an area for further investigation.

### Future direction

Future research should focus on long-term follow-up and the assessment of performance on actual patients. There is an urgent need for studies that apply rigorous human factors and user-centered design principles to the development of AI tutors, with the explicit goal of minimizing extraneous cognitive load. Future RCTs should move beyond the simple AI vs. expert dichotomy and explicitly test the effectiveness of "centaur" models, comparing AI-augmented expert instruction against both standalone AI and traditional instruction. Longitudinal studies are required to assess not only immediate skill acquisition but also long-term retention and, most importantly, the successful transfer of skills from the simulator to the operating room. The field must converge on standardized, validated metrics for both performance and cognitive load to facilitate future meta-analyses and build a more cohesive body of evidence. Additionally, consensus work is needed to establish minimal important differences (MIDs) for OSATS scores in novice surgical trainees to better interpret the clinical significance of future findings.

## Conclusion

Our meta-analysis suggests that AI-tutored systems may be a valuable tool in surgical education. Based on a limited number of studies with low to moderate certainty evidence, AI tutors appear to facilitate skill acquisition to a degree comparable to traditional expert instruction. A small, statistically significant advantage for AI was noted in expert-rated skill assessments, though this finding was driven predominantly by a single high-risk-of-bias study and its clinical relevance is uncertain. These potential benefits must be weighed against significant challenges, including the imposition of higher extraneous cognitive load on learners and high variability in effectiveness across different systems. Our findings do not support the simple replacement of human instructors with AI. Instead, the current evidence points toward a potential role for AI as an adjunct tool within a hybrid "centaur" model, though this model itself requires rigorous empirical validation. More high-quality research is urgently needed to optimize AI tutor design and confirm its long-term impact on skill transfer and patient safety.

## Supplementary Information

Supplementary Material 1.

## Data Availability

No datasets were generated or analysed during the current study.

## Abbreviations

AI: Artificial Intelligence
ANN: Artificial Neural Networks
AR: Augmented Reality
CLI: Cognitive Load Index
CNN: Convolutional Neural Network
DNN: Deep Neural Network
GRADE: Grading of Recommendations Assessment, Development and Evaluation
ICEMS: Intelligent Continuous Expertise Monitoring System
IRB: Institutional Review Board
k-NN: K-Nearest Neighbors
LMICs: Low- and Middle-Income Countries
LRCN: Long-Range Recurrent Convolutional Network
LSTM: Long Short-Term Memory
MCID: Minimum Clinically Important Difference
MD: Mean Difference
MES: Mental Effort Scale
MID: Minimally Important Difference
NCT: Non-Randomized Controlled Trial
NOS: Newcastle–Ottawa Scale
OSATS: Objective Structured Assessment of Technical Skills
OSCE: Objective Structured Clinical Examination
PICOS: Population, Intervention, Comparator, Outcomes, Study Design;
PRISMA: Preferred Reporting Items for Systematic Reviews and Meta-Analyses
PROSPERO: International Prospective Register of Systematic Reviews
RCT: Randomized Controlled Trial
ResNet: Residual Neural Network
RF: Random Forest
RoB 2: Cochrane Risk of Bias 2
SVM: Support Vector Machine
VR: Virtual Reality
