# Supplementary material for: Artificial intelligence augmented tutoring vs expert instruction on learning simulated general surgical skills: a systematic review and meta-analysis
Source: BMC Med Educ. 2026 Jun 10;26:952. doi: 10.1186/s12909-026-09606-9 (PMC13251200; doi:10.1186/s12909-026-09606-9)
Supplement: Supplementary file 1 — Supplementary Material 1. [file 12909_2026_9606_MOESM1_ESM.zip › Search strategy .docx]

| Databases | Search Strategy for Each Database |  |
| --- | --- | --- |
| PubMed  Web of Science  Scopus  Cochrane | ((Artificial Intelligence OR AI OR artificial intelligence OR ChatGBT OR intelligent tutoring system )) AND ((Education OR Teaching OR Tutoring OR Instruction OR Teaching OR Education OR Learning OR Training OR Computer-Assisted Instructions)) AND ((Surgical Procedures Operative OR surgical skill OR surgical train OR surgical educat OR surgical simulation OR procedural skill OR surgical performance OR operative skill )) | 9966  1203  6925  40 |
| Embase  Ovid (Midline) | ("Artificial Intelligence" OR "AI" OR "artificial intelligence" OR "ChatGBT" OR "intelligent tutoring system")  AND ("Education" OR "Teaching" OR "Tutoring" OR "Instruction" OR "Learning" OR "Training" OR "Computer-Assisted Instruction*") AND ("Surgical Procedures Operative" OR "surgical skill*" OR "surgical train*" OR "surgical educat*" OR "surgical simulation" OR "procedural skill*" OR "surgical performance" OR "operative skill*") | 780  493 |

Table 1 shows the search strategy that will be used for each specific database up to August 2025; a total of 19,407 after removing duplicates, 15,594 uploaded to Rayyan
