# Supplementary figures and images for: Artificial intelligence augmented tutoring vs expert instruction on learning simulated general surgical skills: a systematic review and meta-analysis
Source: BMC Med Educ. 2026 Jun 10;26:952. doi: 10.1186/s12909-026-09606-9 (PMC13251200; doi:10.1186/s12909-026-09606-9)

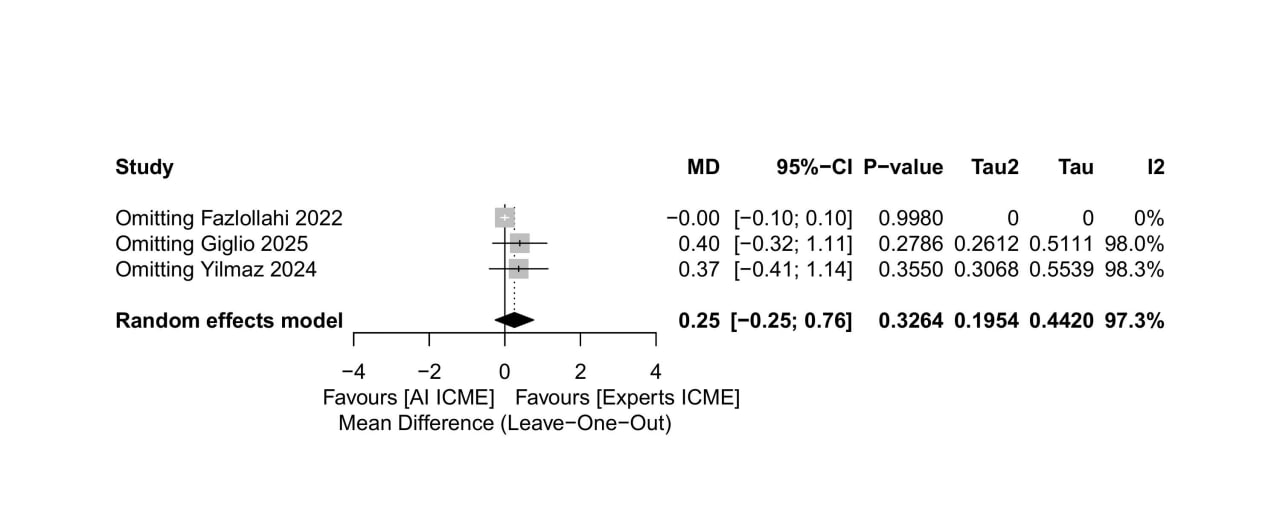

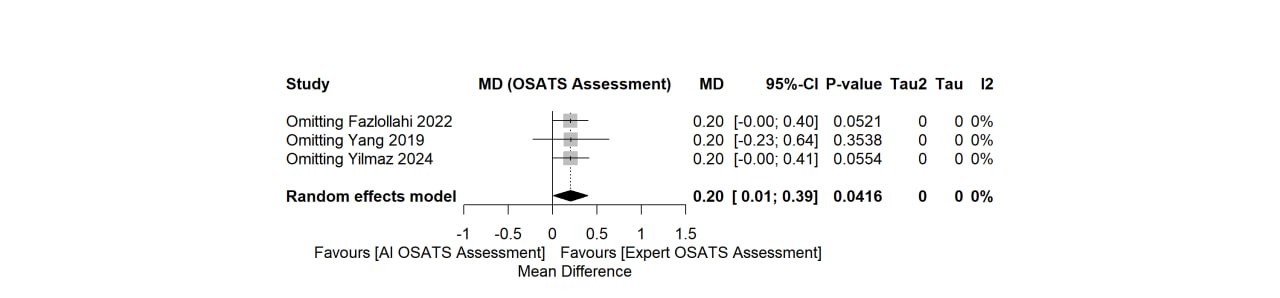
Leave one out analysis results


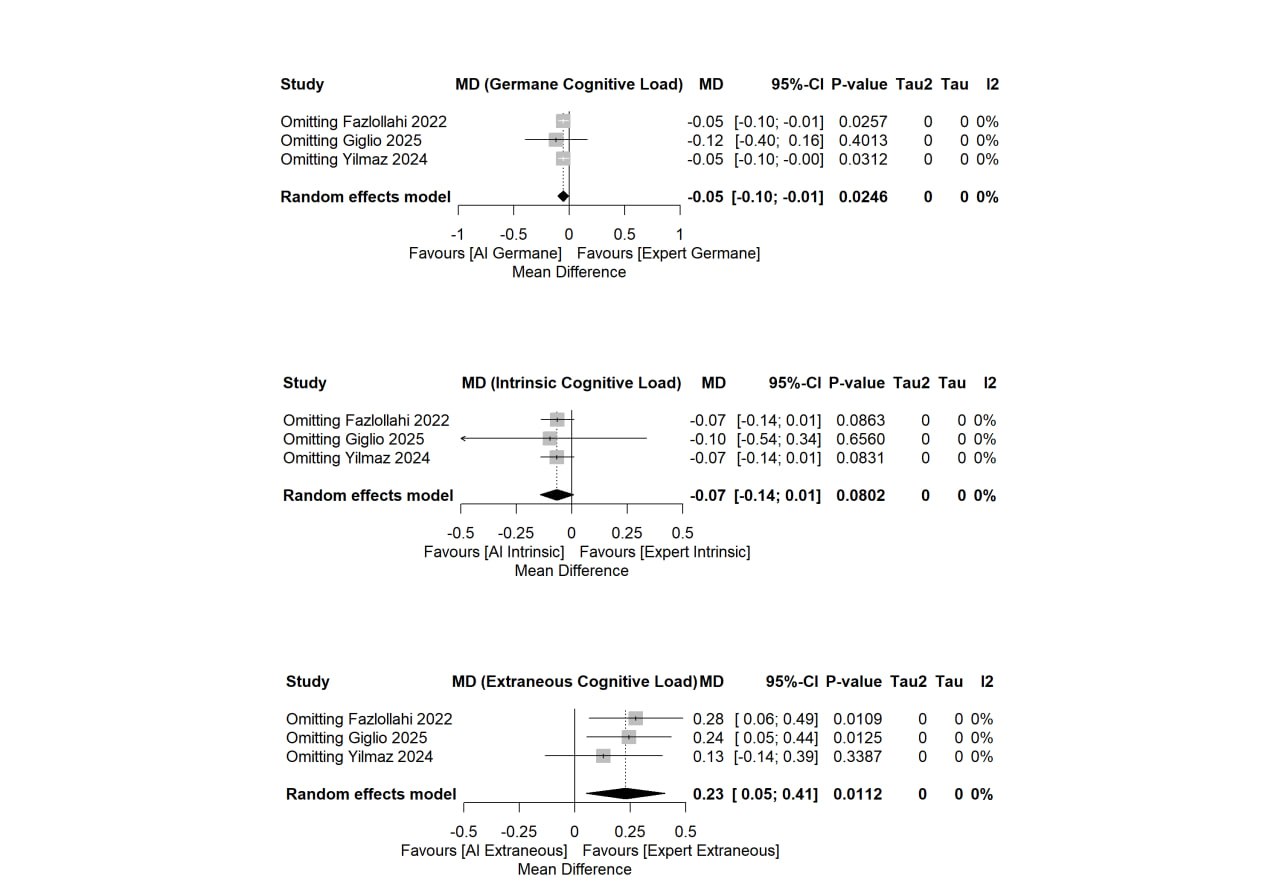

Supplement: Supplementary file 1 — Supplementary Material 1. [file 12909_2026_9606_MOESM1_ESM.zip › ESM 3.docx]

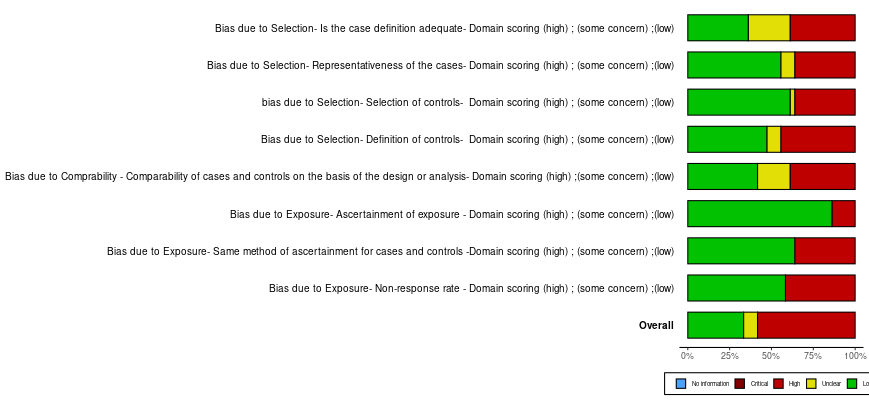

Supplement: Supplementary file 1 — Supplementary Material 1. [file 12909_2026_9606_MOESM1_ESM.zip › NOS summary.png]
